# Short-term All-cause In-hospital Mortality Prediction by Machine Learning Using Numeric Laboratory Results

**DOI:** 10.31662/jmaj.2022-0206

**Published:** 2023-09-29

**Authors:** Gen Shimada, Rumi Nakabayashi, Yasuhiro Komatsu

**Affiliations:** 1 Hernia Center, St. Luke’s International Hospital, Tokyo, Japan; 2 Department of Gastroenterological and General Surgery, St. Luke’s International Hospital, Tokyo, Japan; 3 Department of Nephrology, St. Luke’s International Hospital, Tokyo, Japan; 4 Department of Healthcare Quality and Safety, Graduate School of Medicine, Gunma University, Gunma, Japan

**Keywords:** Critical value list, machine learning, mortality prediction, laboratory test

## Abstract

**Introduction:**

A critical value (or panic value) is a laboratory test result that significantly deviates from the normal value and represents a potentially life-threatening condition requiring immediate action. Although notification of critical values by critical value list (CVL) is a well-established method, their contribution to mortality prediction is unclear.

**Methods:**

A total of 335,430 clinical laboratory results from 92,673 patients from July 2018 to December 2019 were used. Data in the first 12 months were divided into two datasets at a ratio of 70:30, and a 7-day mortality prediction model by machine learning (eXtreme Gradient Boosting [XGB] decision tree) was created using stratified random undersampling data of the 70% dataset. Mortality predictions by the CVL and XGB model were validated using the remaining 30% of the data, as well as different 6-month datasets from July to December 2019.

**Results:**

The true results which were the sum of correct predictions by the XGB model and CVL using the remaining 30% data were 61,535 and 61,024 tests, and the false results which were the sum of incorrect predictions were 5,492 and 6,003, respectively. Furthermore, the true results with the different datasets were 105,956 and 102,061 tests, and the false results were 6,052 and 9,947, respectively. The XGB model was significantly better than CVL (*p* < 0.001) in both datasets.

The receiver operating characteristic-area under the curve values for the 30% and validation data by XGB were 0.9807 and 0.9646, respectively, which were significantly higher than those by CVL (0.7549 and 0.7172, respectively).

**Conclusions:**

Mortality prediction within 7 days by machine learning using numeric laboratory results was significantly better than that by conventional CVL. The results indicate that machine learning enables timely notification to healthcare providers and may be safer than prediction by conventional CVL.

## Introduction

A critical value is “*a pathophysiological state at such variance with normal as to be life threatening unless something is done promptly and for which some corrective action could be taken*” ^(1)^. According to the Joint Commission International Accreditation Standards for Hospitals, a critical value is defined as* “a variance from normal range that represents a pathophysiologic state that is high-risk or life-threatening, is considered urgent or emergent in nature, and in which immediate medical action is likely necessary to preserve life or prevent a catastrophic occurrence*” ^(2)^. The critical value list (CVL) is used by defining a threshold range for each test item ^(3)^. For example, if a serum potassium value deviates from the threshold range, it is checked by a laboratory technician and reported to the ordering healthcare provider as a critical value due to the increased risk of life-threatening arrhythmia and death. This reporting system, so-called callback system, plays an important role in medical safety by preventing delays in medical care related to unnoticed critical values.

Several scoring systems have been proposed and used to predict various life-threatening conditions, including the Acute Physiology And Chronic Health Evaluation II ^(4)^, which is used to predict mortality in intensive care; Simplified Acute Physiology Score (SAPS II) ^(5)^; Sepsis-related Organ Failure Assessment score ^(6)^; and National Early Warning Score 2 ^(7)^, which predicts sudden clinical deterioration of hospitalized adult patients.

In recent years, machine learning has been used to triage patients presenting to the emergency room ^(8), (9)^ and to predict the deaths of patients admitted to the intensive care unit (ICU) ^(10)^, patients with sepsis ^(11)^, and hospitalized patients ^(12), (13)^. However, there is a lack of a sufficient system to ensure that critical values are presented in a timely manner to the medical professionals who can take action.

Detection of critical value results with high accuracy, which is a trigger for the already-established clinical laboratory callback system, could contribute to both medical safety and operational efficiency. In this study, we developed a new short-term mortality risk prediction model, based on machine learning, and examined its feasibility.

## Materials and Methods

This study was approved (research number: 21-R018) by the institutional review board of St. Luke’s International University.

Prior to the start of the trial, an opt out was posted on the hospital’s website to ensure that enrolled subjects had the opportunity to opt out of participating in the trial.

### Subjects

One hundred ten thousand and sixty-two patients who underwent laboratory testing at an urban single teaching hospital and outpatient clinic, from July 2018 to December 2019, were included. Eligible data were electronically extracted retrospectively from the laboratory information system (LIS) and electronic medical record system. From the LIS, numerical test results were included, whereas tests of patients below 18 years old, tests conducted for general check-up purposes, results with no clinical significance such as constants or lot numbers, and tests conducted only once during the study period were excluded. Mortality information was obtained from the electronic medical record system. Ultimately, 86,720 patients, 335,430 tests, 1,146 unique item types, and 11,581,169 test items were included in the analysis.

### Methods

#### Data preparation

Model data from July 2018 to June 2019 were used for the model creation and test, and unseen data from July to December 2019 were used for external validation.

#### Outcome labels and variables

Tests conducted within 7 days of death were defined as positive, whereas tests conducted more than 8 days before death and those for patients with no record of death were defined as negative and used as outcome labels.

After outliers were excluded using boxplot rules, a value summary list was created for value scaling from model data. The first quartile (Q1) and third quartile (Q3) were calculated from the measured values for each of the 1,146 unique item types. Then, the mean and standard deviation of each item type were calculated with measured values between Q1 − (1.5 × (Q3 − Q1)) and Q3 + (1.5 × (Q3 − Q1)) (Supplement A. Value summary list).

If an actual test value of a test item *k* on a test date *j* for a patient *i* was *X_ijk_*, the mean of the test items in the value summary list was *μ_k_*, and the standard deviation was *σ_k_*, *SDL_ijk_* was calculated as follows:


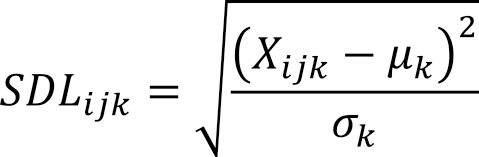


When *SDL_ijk_* equaled or exceeded 2, it was defined as *Over2SD_ijk_*, and when it equaled or exceeded 3, it was defined as *Over3SD_ijk_*.


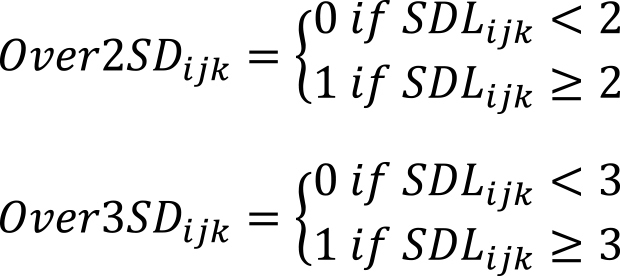


For each examination date, 44 variables and one outcome label were created: gender, age at examination, department requesting examination, patient location (ICU, general ward, palliative care unit and outpatient), presence of dialysis, number of examination items, sum of Over2SD, sum of Over3SD, ratio of sum of Over2SD to number of examination items, ratio of sum of Over3SD to number of examination items, and mean SDL. No missing values were generated during the calculation process.

#### Model creation

The model data were stratified and randomly divided at a ratio of 70:30, and then stratified random undersampling was performed on 70% of the data to be used as the training data. A gradient boosting decision tree (XGB) model was created using the receiver operating characteristic-area under the curve (ROC-AUC) as the evaluation metrics, and 10-fold cross-validation was performed. The model was tuned by random grid search and finalized. The software and library used were Python 3.8.10, pandas 1.3.5, imbalanced-learn 0.7.0, Matplotlib 3.5.1, NumPy 1.19.5, scikit-learn 0.23.2, and SciPy 1.5.4.

#### Test and external validation

The results of machine learning (XBG) prediction and CVL (Table 1) were compared on two datasets: unused 30% of the model data and unseen external validation data. The results were compared using a confusion matrix and ROC curves.

**Table 1.** Critical Value List (Numeric Range Only).

Category	Test item	Critical value
Chemistry	Glucose	≦50 or ≧450 mg/dL
	Creatinine	≧3 mg/dL
	Total bilirubin	≧5 mg/dL
	Alkaline phosphatase	≧1000 IU/L
	Lactate dehydrogenase	≧1000 IU/L
	Aspartate aminotransferase	≧1000 IU/L
	Alanine aminotransferase	≧1000 IU/L
	Amylase	≧1000 IU/L
	Creatine kinase	≧5000 IU/L
	Calcium	≦6 or ≧12 mg/dl
	Sodium	≦119 or ≧160 mmol/L
	Potassium	≦2.5 or ≧6 mmol/L
Hematology	White blood cell count	≦2 or ≧20 × 10^3^/mm^3^
	Hemoglobin	≦8 or ≧17 g/dL
	Platelet count	≦50 or ≧700 × 10^3^/mm^3^
	Neutrophil count	≦0.5 × 10^3^/mm^3^
	Lymphocyte rate	≧70%
	Atypical lymphocyte rate	≧10%
	Eosinocyte rate	≧20%
Coagulation	PT-INR	≧4
	Activated partial thromboplastin time	≧225 seconds
	Fibrinogen	≦100 mg/dL

PT-INR: Prothrombin time-international normalized ratio

The true and false results were defined as the sum of true positives and true negatives and the sum of false positives and false negatives, respectively. Fisher’s exact test was used to test the XGB and CVL results. SHAP (SHapley Additive exPlanations) ^(14)^ was used to interpret the models.

## Results

The data comprised 223,422 tests as the model data and 112,008 tests as the external validation data. The positive outcome label was 1,334/223,422 (0.6%) for the model data and 589/112,008 (0.5%) for the validation data (Table 2). Because 70% of the stratified random split of the model data was unbalanced data with 934/155,461 (0.6%) positives, stratified random undersampling was performed to obtain the training data with 934/1,868 (50%) positives.

**Table 2.** Cohort Characteristics.

	Model data	External Validation data
Period	12 months	6 months
Number of patients	51240	35480
Gender		
Male	21031	15118
Female	30209	20362
Age at laboratory tests (mean ± SD)	59.0 ± 18.5	59.1 ± 18.5

Number of laboratory tests	223422	112008
Number of laboratory test items	7691243	3889926

Number of laboratory tests by patient location		
Outpatient	169743	84773
General ward	42089	21793
Intensive care unit	10944	5155
Palliative care unit	646	287

Number of laboratory tests by requesting department		
Obstetrics and gynecology	36946	18105
Immuno-Rheumatology	22013	11000
Cardiology	20785	10208
Nephrology	15464	8158
Emergency room	12044	6181
Urology	11815	5731
Endocrinology	11772	6002
Gastroenterology	11509	5999
Hematology	10493	5036
Anesthesiology	10110	5012
Oncology	10004	5188
Respiratory medicine	9351	4795
Internal medicine	9177	4569
Breast surgery	8480	3680
General surgery	6167	3157
Infectious disease	5821	2651
Cardiovascular surgery	4518	2668
Orthopedics	4423	2310
Dermatology	3345	1685
Neurosurgery	3114	1814
Neurology	2313	1041
Psychiatry	1329	729
Thoracic surgery	1324	765
Ophthalmology	1158	566
Plastic surgery	942	499
Neurovascular	774	275
Palliative care	693	312
Otorhinolaryngology	686	412
Oral surgery	212	123
Radiology	156	43
Others	373	264

Number of laboratory tests with critical values	20297	9948
Number of deceased patients	522	260
Number of laboratory tests conducted within 7 days of death	1334 (0.6%)	589 (0.5%)

The cross-validation results during modeling are presented in Table 3. The ROC-AUC and Recall values were stable and high, resulting in 0.9796 ± 0.0053 and 0.9540 ± 0.0234, respectively. Meanwhile, their precision was low at 0.0642 ± 0.0049. Thus, F1 was calculated as 0.1203 ± 0.0087.

**Table 3.** Cross-Validation Result.

Validation	Accuracy	ROC-AUC	Recall	Precision	F1
1	0.9197	0.9757	0.9355	0.0651	0.1217
2	0.9222	0.9839	0.9787	0.0704	0.1313
3	0.9113	0.9688	0.9255	0.0593	0.1114
4	0.9133	0.9838	0.9681	0.0630	0.1183
5	0.9072	0.9750	0.9255	0.0568	0.1071
6	0.9112	0.9811	0.9785	0.0616	0.1158
7	0.9195	0.9849	0.9785	0.0675	0.1263
8	0.9261	0.9868	0.9785	0.0732	0.1361
9	0.9190	0.9793	0.9462	0.0652	0.1220
10	0.9134	0.9768	0.9247	0.0600	0.1127
**Mean ± SD**	**0.9163 ± 0.0056**	**0.9796 ± 0.0053**	**0.9540 ± 0.0234**	**0.0642 ± 0.0049**	**0.1203 ± 0.0087**

Test and external validation summary and prediction results are presented in Table 4.

**Table 4.** Summary and Prediction Results from 30% Model Data and Validation Data.

	30% of Model data		Validation data	
Number of patients	29258		35480	
Number of deceased patients	270		260	
Number of laboratory tests	67027		112008	
Number of negative laboratory tests	66627		111419	
	Correct	Incorrect	Correct	Incorrect
CVL prediction	60785	5842	101754	9665
XGB prediction	61153	5474	105496	5923
Number of positive laboratory tests	400		589	
	Correct	Incorrect	Correct	Incorrect
CVL prediction	239	161	307	282
XGB prediction	382	18	460	129

Negative laboratory tests were tests conducted on survivors more than 7 days before death. Positive laboratory tests were tests conducted within 7 days before death. CVL: Critical value list XGB: Gradient boosting decision tree prediction

The 400 positive laboratory tests performed within 7 days of death in the 30% model data were performed on 270 deceased patients. Similarly, 589 positive laboratory tests in the validation data were conducted on 260 patients.

In the 30% model data, CVL correctly predicted 60785 (91.2%) of 66627 negative results and failed to correctly predict 5842 of them. Furthermore, CVL correctly predicted 239 (59.8%) of 400 positive results and failed to correctly predict 161. The ROC-AUC, Recall, precision, and F1 values resulting from CVL prediction were 0.7549, 0.5975, 0.0393, and 0.0738, respectively.

XGB correctly predicted 61,153 (91.8%) of the negative results and 382 (95.5%) of the positive results. The ROC-AUC, Recall, precision, and F1 values resulting from 30% model data prediction were 0.9805, 0.9550, 0.0652, and 0.1221, respectively.

In the external validation data, CVL and XGB predicted 10,1754 (91.3%) and 10,5496 (94.7%) of the 111,419 negative results and 307 (52.1%) and 460 (78.1%) of the 589 positive results, respectively. The ROC-AUC, Recall, precision, and F1 values resulting from the external validation data prediction were 0.9702, 0.8964, 0.0574, and 0.1078, respectively.

A comparison of CVL and XGB with the 30% model data and the external validation data is presented in Table 5.

**Table 5.** Comparison of Prediction Results from 30% Model Data and External Validation Data.

	30% of Model data			External Validation data		
	True result	False result	p	True result	False result	p
CVL result	61024	6003	<0.001	102061	9947	<0.001
XGB result	61535	5492		105956	6052	

True result was the sum of correct predictions among all the laboratory tests. False result was the sum of incorrect predictions among all the laboratory tests. CVL: Critical value list XGB: Gradient boosting decision tree

The true results which predicted positive and negative correctly for CVL and XGB were 61,024 and 61,535 tests in the 30% model data, respectively. The false results which predicted incorrectly for CVL and XGB were 6,003 and 5,492 tests, respectively. XGB had significantly improved prediction ability compared with CVL in Fisher’s exact test (*p* < 0.001).

The same results were confirmed by the external validation data. The true results for CVL and XGB were 102,061 and 105,956 tests and the false results were 9,947 and 6,052 tests, respectively.

Comparison of CVL and XGB using the validation data showed that XGB was significantly predictive with true and false result (*p* < 0.001).

**Figure 1.**
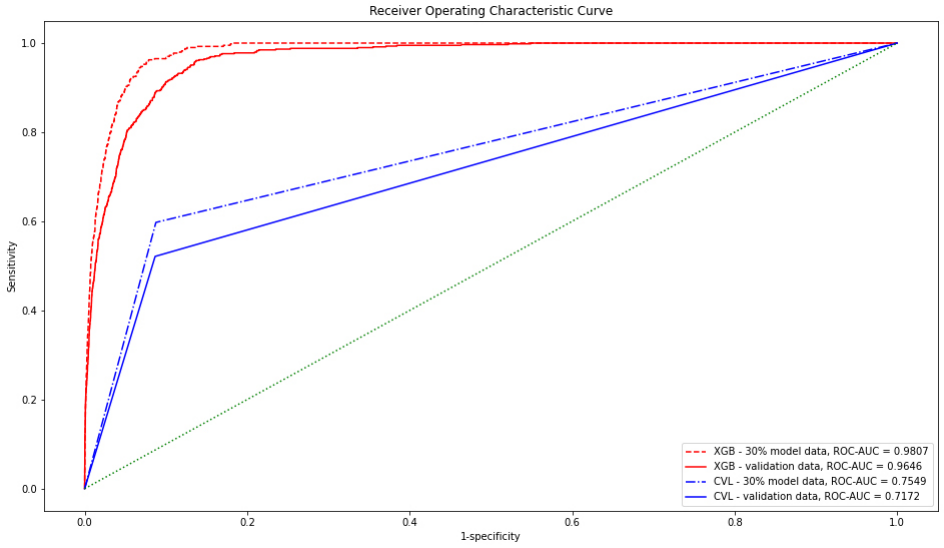
Receiver Operating Characteristic Curve CVL: critical value list prediction XGB: gradient boosting decision tree prediction ROC-AUC: receiver operating characteristic–area under the curve.

**Figure 2.**
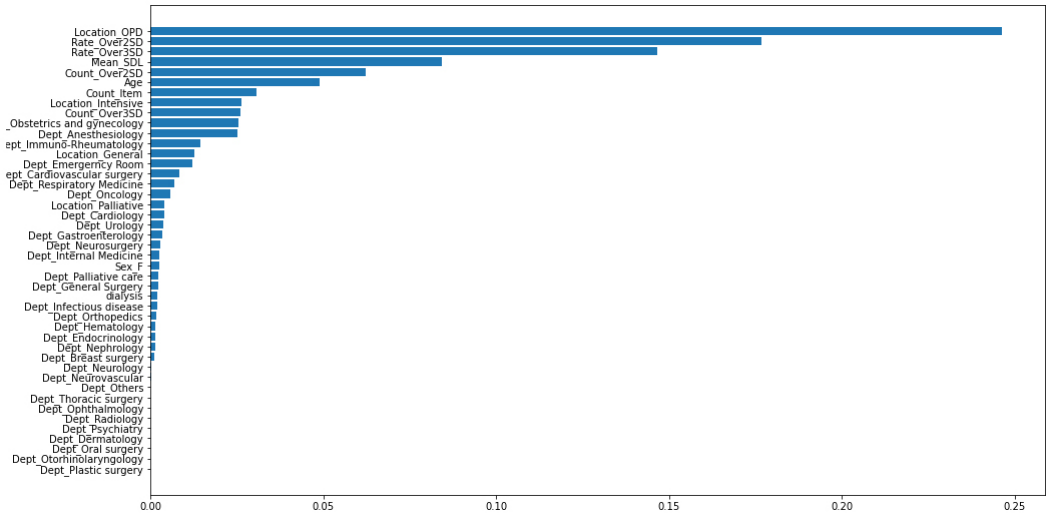
Future importance.

Figure 1 presents the ROC-AUC for the 30% and external validation data by XGB, which were 0.9807 and 0.9646, much higher than 0.7549 and 0.7172 by CVL, respectively. Figure 2 shows the variables that contributed to the model in the decreasing order of feature importance: outpatient department, rate of over 2 SD, rate of over 3 SD, mean SDL, and count of over 2 SD.

**Figure 3.**
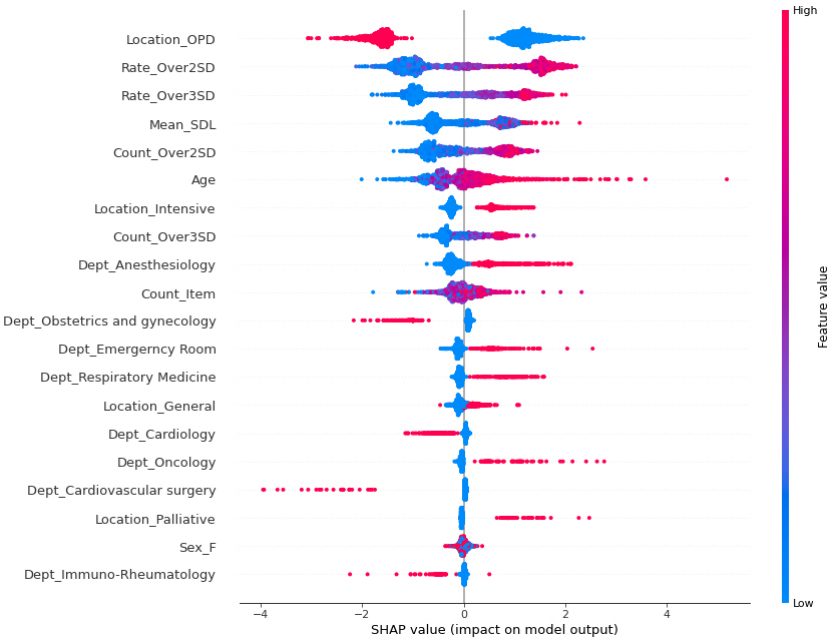
SHAP result.

The SHAP results for each variable are presented in Figure 3. When Location OPD = 1, i.e., the feature value is high, and the SHAP value indicates minus; conversely, when Location OPD = 0, i.e., the feature value is low, and the SHAP value indicates plus. These results suggest that the impact was negative for outpatients and positive for nonambulatory patients. Meanwhile, higher values for the following variables showed a positive impact: rate of over 2 SD, rate of over 3 SD, count of over 2 SD, count of over 3 SD, ICU, palliative care unit, and oncology department.

## Discussion

Critical values are laboratory values that significantly deviate from normal values, such as serum potassium and blood glucose levels, and represent pathological conditions that could be life-threatening if immediate action is not taken.

A system has been installed whereby laboratory personnel who identify critical values verify their accuracy and notify the pertinent persons, by their own responsibility, so that appropriate action can be taken. This system is widely recognized as a mechanism to enhance medical safety ^(15)^ and is one of the certification requirements in the Joint Commission International Accreditation Standards for Hospitals ^(2)^.

ISO 15189:2012 (Medical laboratories - Requirements for quality and competence) defines alert interval and critical interval as “interval of examination results for an alert (critical) test that indicates an immediate risk to the patient of injury or death” and noted “the laboratory determines the appropriate list of alert tests for its patients and users.”

A general list of critical values was presented by the Emancipator based on the results of a survey conducted on several medical facilities, but it stated that it would be counterproductive to attempt to implement a uniform list for all laboratories and that each laboratory must customize its list to meet the needs of its organization ^(3)^. For this reason, CVLs are currently being used by individual laboratories to set thresholds for a limited number of laboratory items. Furthermore, the CVL covers only a single test item and score values, such as those obtained by combining multiple test items, which are rarely the subject of expedited reporting.

There is limited evidence to define critical value thresholds. This is due in part to the difficulty of designing clinical trials to investigate the clinical outcomes of different threshold test results. It is ethically unacceptable to study the clinical outcomes of patients with critical values while withholding treatment. Therefore, the list of critical values varies ^(16), (17), (18), (19), (20)^.

Meanwhile, it has been proposed to create a CVL using measured values with a 90% probability of death within 24 h if left untreated ^(21)^ or using Bayesian theory to calculate critical outcome thresholds for adult deceased patients ^(22)^ rather than on a consensus basis threshold.

Improper definition of critical value thresholds may lead to increased over- and undernotification. The former may cause alert fatigue, whereas the latter may pose a risk of delaying timely treatment to vulnerable patients. In addition, a large outlier on a test that is not on the CVL will not trigger the callback system and may go unnoticed. In either case, safety is threatened.

In our study, we created our own variables using mainly numerical test results, not limited to a list of critical values, and used machine learning to predict mortality within 7 days of the test date for adult patients. In addition, rather than using the numerical test values, we normalized the difference from the mean by standard deviation for each test item and included variables such as the percentage of test items with two or more standard deviations away from the mean of the number of items in the test conducted on a single day. One of the strengths of this method was that the results of the majority of other tests can be used in the entire period except for the one-time-only tests. Other strengths were that no missing values were generated and that only data within the LIS was required to use this method.

The exclusion of test value outliers is aimed at creating a value summary list. This list is solely utilized for the transformation of actual test values. In data preprocessing, the list is only used for scaling numeric test values, and there is minimal risk of information loss.

Literatures of mortality prediction using machine learning demonstrate that the mortality prediction rate was improved by using clinical information such as vital signs, medical records, and routine-specific examinations at the time of admission ^(12), (23), (24)^. However, there were many challenges in real-time prediction due to the data cleaning and preprocessing steps and the occurrence of missing values when routine tests were not conducted or when medical records were delayed ^(24), (25)^. By not using data outside of the LIS, such as images, vital signs, and clinical notes, it is unnecessary to extract or link data from other systems, such as electronic medical records and PACS, and to wait for the results of these systems and consider missing values. Thus, it is possible to make predictions when the test results within the LIS become available, leading to minimum time lag.

Machine learning with gradient boosting decision tree, which was used in this study, has been employed in previous studies and has shown high prediction performance ^(23), (25), (26), (27)^.

In the data collected in this study, the numbers of tests conducted within 7 days of death were 0.06% and 99.94% for positive and negative cases, respectively, indicating a large imbalance in the frequency of the event to be determined. Machine learning classification models are generally designed to be most accurate for balanced data, in which the desired event is 1:1. Machine learning prediction for unbalanced data is known to improve accuracy by learning after appropriate sampling ^(28), (29)^. Class imbalance is one of the most common scenarios in real-world setting. Various methods, such as random undersampling, random oversampling, synthetic data generation techniques such as SMOTE (Synthetic Minority Oversampling TEchnique), or class-weighting, have been proposed to address class imbalance and improve model prediction. Before this study, we conducted a preliminary investigation using oversampling, SMOTE, and class-weighting to mitigate the imbalance issue. As a result, both oversampling and SMOTE led to a significant increase in data volume, which consumed a significant amount of time for model training and tuning. Similarly, class-weighting also required additional training time. However, none of these methods showed improved prediction compared with random undersampling. Due to the fact that positive instances (death cases) were in the minority and the negative instances were abundant, and considering the need to reduce the training and tuning times, we applied random undersampling on the negative instances containing a large number of normal tests. In this study, addressing class imbalance using stratified random undersampling is considered practical, despite the potential impact on the model’s performance and result interpretation, as well as the inclusion of inherent bias.

The ROC-AUC for predicting deaths within 7 days is extremely high, exceeding 0.95. It is noteworthy that this accuracy was achieved not only in the ER, intensive care, and palliative care areas, where many patients are critically ill or have died, but also in general wards and outpatient departments, where deaths are rare. Furthermore, the ROC-AUC exceeded 0.95 in the external validation results using data from different time periods, indicating the reliability of this method.

The ROC-AUC and Recall show high values, indicating that the model performs well in identifying positive cases. However, the low precision and F1 score indicate the presence of many false positives. Consequently, complete automation and direct notification to the clinician may not be appropriate due to the risk of unnecessary interventions or tests caused by the false positive. Meanwhile, allowing for a false negative in short-term mortality prediction may result in some necessary interventions or tests not being conducted, making it an unsafe option from a clinical perspective.

As a solution, in addition to the CVL policy established for the callback system, clinical laboratory technicians can use this model prediction as additional reference to deliver more accurate critical value callback to the clinicians. This approach allows leveraging the expertise and experience of the technicians to make more reliable judgments.

The fact that both false positives and false negatives were reduced compared with the CVL indicates that alert fatigue was reduced and life-threatening patients were picked up more specifically, which is clearly more beneficial than the current situation. This is considered to contribute to the improvement of medical safety.

The Feature Importance plot is presented in Figure 2. The features that contributed to the prediction are listed in the order of their importance, starting with whether the examination was conducted in an outpatient setting or not, followed by Rate_Over2SD, Rate_Over3SD, Mean_SDL, Count_Over2SD, Age, Count_Item, and Location_Intensive.

The Feature Importance plot provides an understanding of the significant features that contribute to the prediction. However, it does not show how the values of these features specifically influence the results. Further analysis or interpretation is warranted to understand how the values of these features contribute to the predictions in different scenarios.

The SHAP results presented in Figure 3 suggest that the SHAP value, which indicates the impact on the model outcome, is lower when the feature value, i.e., 1, is high for Location_OPD (outpatient), and when the feature value is higher when Location_OPD = 0, i.e., the patient is not an outpatient. The feature value is higher when Location_OPD = 0, i.e., not an outpatient, and the model has more deaths because the SHAP value is over 0. When Rate_Over2SD (rate of over 2 SD), Rate_Over3SD (rate of over 3 SD), and Mean_SDL(mean of SDL) are higher SHAP values.

Furthermore, it has been demonstrated that SHAP values are particularly high for cases with certain characteristics, such as being elderly and having specific medical conditions, such as Anesthesiology, Emergency Medicine, Respiratory Medicine or Oncology, as well as being in specific locations such as the ICU or palliative care unit. This suggests a higher number of short-term mortality cases among patients with these characteristics.

The anesthesiology department is in charge of intensive care in our hospital. As a result, there are many blood test requests for ICU patients coming from this department. Higher SHAP values are attributed to the characteristics of the anesthesiology department.

Based on the overall interpretation of the SHAP plot, higher SHAP values are observed in the following scenarios: 1. non-outpatient cases: patients who are admitted as inpatients show higher SHAP values; 2. higher daily number of tests: patients who undergo several tests per day exhibit higher SHAP values; 3. significant outliers in test results: patients with a higher proportion of test results exceeding 2 or 3 standard deviations from the threshold have higher SHAP values; 4. elderly patients: SHAP values tend to be higher in older patients and ICU and palliative care units: patients admitted to the ICU and palliative care units show higher SHAP values; and 5. specific medical conditions: patients with medical conditions related to emergencies, respiratory issues, or malignant tumors have higher SHAP values. These factors seem to contribute to higher SHAP values in the model and can be important indicators of patient outcomes. These results fit well with the sense of daily practice.

Our method, which uses inspection results expressed as numerical results, contains several strengths. First, many test items not covered by the CVL can be used as variables. Second, its use is not restricted to situations in which medical care is provided, such as outpatient and intensive care settings. Third, the model can be created from existing data and does not require new data. Fourth, it does not require data outside the LIS, such as electronic medical records or PACS images. From the perspective of integration into the existing system, because the LIS system handles everything internally, the actual implementation barrier is expected to be low. Once the laboratory results are saved within the LIS system, this predictive model becomes available for use. In data preprocessing, test value scaling and aggregation of these values are necessary. Considering the number of test items conducted per patient per day, the generation of predictions is almost real time, thus minimizing time lag and cost for connection to other information systems. Finally, the LIS used in medical practice already incorporates appropriate data privacy and information security. Therefore, when using this model within the LIS environment, there is little to no impact on existing data privacy and information security. Because no new information integration to electronic medical record system or PACS is required, and this model operates within the existing framework of the LIS, the risks to data privacy and information security are kept to a minimum.

Because the results of specific test items are not required, there is no need to add or modify test items that are currently performed routinely or that are unique to specialized departments. In other words, this system is suggested to help improve medical safety without changing current practice patterns.

The challenges in this study arise from the attempt to predict short-term mortality using mainly clinical laboratory results, and it is fundamentally impossible to accurately predict short-term mortality solely based on this dataset. This limitation is due to the exclusion of items outside the value summary list, qualitative results, and patients who did not undergo laboratory sampling, making it impossible to correctly predict the mortality of all patients. Furthermore, relying mainly on a limited dataset makes it difficult to accurately distinguish between survival and mortality. In other words, even with the same features, there is a mixture of surviving and deceased patients, leading to data uncertainty. As a result, increasing the amount of training data alone does not significantly improve predictive accuracy. Consequently, the precision, F1 score, and false positives in the results of this study remain low.

One proposed solution to address this challenge is to incorporate additional domain knowledge, such as vital signs, images, medical records, treatment details, and clinical courses, into the model. However, the use of these data introduces new challenges, such as dealing with issues of timing, documentation accuracy by clinicians, data integration, handling of missing values, and trigger timing.

This research aimed to enhance mortality prediction using the current CVL. It is necessary to make predictions at the time of CVL evaluation. Thus, the addition of new domain knowledge to address data uncertainty was not pursued. Nevertheless, achieving a significantly higher predictive accuracy than CVL using a model based on limited information is important.

Quantifying the uncertainty of machine learning predictions is essential for the safe use of machine learning predictions. While four methods (single deterministic, Bayesian, ensemble, and test-time augmentation methods) have been proposed for quantifying prediction uncertainty in deep neural networks ^(30)^, the quantification of prediction uncertainty in XGB models is limited and was not included in this study.

The reason is that, due to the nature of this study, the following challenges are significantly involved in the uncertainty of predictions. These challenges include data uncertainty, domain-shift uncertainty due to changes in the target patient population or severity, and addressing out-of-domain uncertainty resulting from the introduction of completely new laboratory test or changes in testing items.

In the external validation with a different time period, significant differences in prediction results for this model were not observed. However, when continuously using the model, monitoring and updating of the value summary list and regular retraining are considered crucial.

This model has several limitations. Prediction cannot be made without a laboratory test as laboratory numeric results are required. Furthermore, the value summary list needs to be routinely updated when a new test is installed. The accuracy of the model may be lower for hematology and dialysis patients whose test results contain many abnormalities despite their conditions being relatively stable. In addition, this is a study conducted retrospectively within one facility. Thus, it may include selection bias or limit the control over potential confounding factors. It is necessary to verify our results prospectively also at other institutions.

### Conclusion

Compared with the CVL, machine learning using blood examination results was able to predict short-term mortality with extremely high accuracy, reducing both false positives and false negatives. This method suggests that rapid clinical feedback can contribute to appropriate patient care and further medical safety.

## Article Information

### Conflicts of Interest

None

### Acknowledgement

We thank Sachiko Ohde (Graduate School of Public Health, St. Luke’s International University) for her statistical advice.

### Author Contributions

Acquisition of data: GS, RN

Drafting of the manuscript: GS, YK

Revision of the manuscript for important intellectual content: GS, RN, YK

Supervision: YK

All authors approved the manuscript to be published and agree to be accountable for all aspects of the work in ensuring that questions related to the accuracy or integrity of any part of the work are appropriately investigated and resolved.

### Approval by Institutional Review Board (IRB)

This study was performed as 21‐R018 approved by the institutional review board of St. Luke’s International University.

## Supplement

Supplementary Materials
