# Supplementary material for: Short-term All-cause In-hospital Mortality Prediction by Machine Learning Using Numeric Laboratory Results
Source: JMA J. 2023 Sep 29;6(4):470–80. doi: 10.31662/jmaj.2022-0206 (PMC10628331; doi:10.31662/jmaj.2022-0206)
Supplement: Supplementary Materials [file 2433-3298-6-4-470-s001.pdf]

Supplemental Table 1. Confusion matrix of CVL result from 30% model data

	CVL negative	CVL positive
Actual negative	60785	5842
Actual positive	161	239

CVL: critical value list prediction

Supplemental Table 2. Confusion matrix of XGB result from 30% model data

	XGB negative	XGB positive
Actual negative	61153	5474
Actual positive	18	382

XGB: Gradient boosting decision tree prediction

Supplemental Table 3. Comparison of CVL and XGB result from 30% model data

	True result	False result	Fisher's exact test
CVL result	61024	6003	$p < 0.001$
XGB result	61535	5492	

CVL: critical value list prediction

XGB: Gradient boosting decision tree prediction

True result = True positive + True negative

False result = False positive + False negative

Supplemental Table 4. Confusion matrix of CVL result from validation data

	CVL negative	CVL positive
Actual negative	101754	9665
Actual positive	282	307

CVL: critical value list prediction

Supplemental Table 5. Confusion matrix of XGB result from validation data

	XGB negative	XGB positive
Actual negative	105496	5923
Actual positive	129	460

XGB: Gradient boosting decision tree prediction

Supplemental Table 6. Comparison of CVL and XGB result from validation data

	True result	False result	Fisher's exact test
CVL result	102061	9947	$p < 0.001$
XGB result	105956	6052	

CVL: critical value list prediction

XGB: Gradient boosting decision tree prediction

True result = True positive + True negative

False result = False positive + False negative

Supplement material A. value summarize list

Analytic code	item name	material	count	mean	sd†
**001003_002**	Hemoglobin	venous blood	179286	12.37913	2.096656
**001002_002**	Red Blood Cell Count	venous blood	178906	4.080003	0.704278
**001004_002**	hematocrit	venous blood	178593	37.25434	5.845528
**001007_002**	mean erythrocyte hemoglobin concentration	venous blood	176968	33.28593	1.018126
**001010_002**	mean platelet volume	venous blood	175900	9.741392	0.837561
**001005_002**	Mean erythrocyte volume	venous blood	173711	91.60695	4.869397
**001009_002**	platelet count	venous blood	173001	225.1249	67.98841
**001006_002**	mean erythrocyte hemoglobin	venous blood	172104	30.57498	1.815188
**001001_002**	White Blood Cell Count	venous blood	171863	6.120542	2.139685
**001008_002**	red blood cell distribution width	venous blood	168527	13.42876	1.27439
**000027_001**	Estimated glomerular filtration rate	Blood	160291	71.0397	24.78968
**000036_001**	Potassium	Blood	153323	4.199041	0.4266
**000005_001**	creatinine	Blood	151697	0.741525	0.225794
**000018_001**	Alanine aminotransferase	Blood	150407	19.76619	9.461018
**000017_001**	Aspartic acid Aminotransferase	Blood	150223	22.64374	7.107569
**000004_001**	urea nitrogen	Blood	145510	15.37456	5.390513
**000019_001**	Aspartate aminotransferase Aspartate aminotransferase Alanine aminotransferase Alanine aminotransferase Ratio	Blood	144461	1.215826	0.406976
**000035_001**	Sodium	Blood	143353	139.8994	2.260509
**000016_001**	Lactate dehydrogenase	Blood	139167	190.4803	41.30296
**000002_001**	albumin	Blood	137736	3.911245	0.579426
**000037_001**	Chlor	Blood	134794	105.0998	2.901648
**000007_001**	Total bilirubin	Blood	129157	0.547835	0.216828
**000015_001**	Alkaline phosphatase	Blood	121796	220.5269	72.77435
**001021_002**	lymphocyte	venous blood	117014	25.42182	11.05261
**001223_002**	Lymphocytes	venous blood	115568	1.406968	0.586916
**001224_002**	Monocytes	venous blood	112192	0.3546	0.140493
**001022_002**	monocyte	venous blood	111518	6.090045	1.871539
**001220_002**	Neutrophil count	venous blood	110953	3.729938	1.727891
**000040_001**	C-reactive protein	Blood	107866	0.455273	0.71215
**000020_001**	γ-Glutamyltransferase	Blood	105990	34.15223	24.15416
**001019_002**	eosinophils	venous blood	103692	2.609196	1.81163
**001221_002**	Eosinophil count	venous blood	102173	0.147172	0.103809
**002541_011**	pH	Fresh Urine	100920	6.255489	0.872041
**002535_011**	Specific gravity	Fresh Urine	98936	1.015856	0.007493
**001020_002**	basophil	venous blood	98830	0.682783	0.33655
**001222_002**	Basophils	venous blood	97604	0.038083	0.018877
**000001_001**	Total Protein	Blood	84769	6.917519	0.574387
**000029_001**	Calcium	Blood	83048	9.166752	0.528777

**001016_002**	Neutrophil	venous blood	82175	61.60124	9.425594
**000025_001**	Creatine kinase	Blood	81457	87.1167	49.33978
**000124_001**	Blood glucose	Blood	76729	110.5822	22.23223
**000003_001**	albumin-total globulin ratio	Blood	71511	1.401986	0.338165
**000006_001**	Uric acid	Blood	68778	5.643418	1.521584
**000014_001**	Neutral Fat	Blood	57047	122.0914	59.22605
**000030_001**	Inorganic phosphorus	Blood	52481	3.479473	0.67289
**000012_001**	HDL-cholesterol	Blood	52379	57.56255	16.19727
**000011_001**	LDL-cholesterol	Blood	49289	107.7727	31.52104
**001109_002**	Prothrombin time activity	venous blood	47312	101.0374	27.79358
**000129_001**	HbA1c	Blood	46736	6.104168	0.687671
**001072_002**	Prothrombin time	venous blood	42500	11.72886	1.512577
**001074_002**	Prothrombin International Normalized Ratio	venous blood	42441	0.973139	0.12391
**001073_002**	Prothrombin time ratio	venous blood	42416	0.97273	0.124197
**000038_001**	Bicarbonate	Blood	41365	25.51859	3.247157
**000022_001**	Amylase	Blood	40434	80.7787	28.7964
**001075_002**	Activated partial thromboplastin time	venous blood	40415	32.09812	5.754972
**001018_002**	nucleus segmentosus neutrophil	venous blood	34908	68.7823	16.99402
**001226_002**	Lobular nucleus neutrophil count	venous blood	34491	5.173451	3.41488
**000031_001**	Magnesium	Blood	34073	2.056461	0.232112
**002596_011**	creatinine	Fresh Urine	31257	84.75961	53.55597
**000010_001**	Total cholesterol	Blood	31016	198.4649	38.80007
**001065_002**	Reticulocyte count	venous blood	28577	0.061442	0.027039
**001211_002**	reticulocyte hemoglobin isovolume	venous blood	27857	33.09381	2.986418
**001066_002**	Reticulocyte	venous blood	27617	1.600349	0.669979
**000039_001**	Anion gap	Blood	27364	8.971258	2.163544
**001069_002**	Erythrocyte Sedimentation Rate 1 hour	venous blood	24657	20.35065	18.18364
**002589_011**	Protein determination	Fresh Urine	24171	12.84196	14.49614
**011278_001**	LDL-Cholesterol	Blood	20647	111.9275	32.50404
**001082_002**	D-dimer	venous blood	18505	3.13471	3.348402
**000059_007**	HCV antibody	Serum	17940	0.1	1.39E-17
**000071_001**	IgG	Blood	17403	1213.21	358.7047
**010103_001**	NT-proBNP	Blood	16506	296.2729	338.7147
**000062_007**	HIV antigen/antibody	Serum	16310	0.124948	0.055313
**000053_007**	HBs antigen	Serum	15334	0.1	1.39E-17
**005043_001**	Thyroid-stimulating hormone	Blood	15143	2.232859	1.554894
**005041_001**	Free thyroxine	Blood	14603	1.270889	0.214939
**001017_002**	Rod-shaped nucleus neutrophil	venous blood	13227	1.136047	0.875466
**001225_002**	Rod-shaped nucleus neutrophil count	venous blood	13142	0.084586	0.075867
**005022_001**	CEA	Blood	12992	2.213347	1.467634
**003064_003**	Hemoglobin	Arterial blood	10697	10.53969	2.125479
**003058_003**	O2CT	Arterial blood	10584	13.92166	2.832903

**003060_003**	Sodium	Arterial blood	10433	138.7825	5.045969
**003061_003**	Potassium	Arterial blood	10344	3.86802	0.5049
**003065_003**	COHb	Arterial blood	10336	1.282914	0.447916
**003067_003**	Blood glucose	Arterial blood	10289	141.2184	39.88834
**003069_003**	mOsm	Arterial blood	10251	285.4928	9.953147
**003062_003**	Chlor	Arterial blood	10248	108.2089	5.154372
**003052_003**	pH	Arterial blood	10227	7.423031	0.053918
**003070_003**	FiO2	Arterial blood	10203	30.04798	10.66676
**003066_003**	FMetHb	Arterial blood	10187	0.83155	0.231315
**003053_003**	Carbon dioxide partial pressure	Arterial blood	10186	37.57244	6.390985
**003055_003**	HCO3	Arterial blood	10185	23.96248	3.749296
**003057_003**	SBE	Arterial blood	10175	0.119332	3.863609
**003056_003**	Actual base excess	Arterial blood	10152	0.274537	3.733085
**003063_003**	Calcium ion	Arterial blood	10123	2.278198	0.116634
**003068_003**	Lactate	Arterial blood	9972	1.172312	0.523049
**003054_003**	Oxygen partial pressure	Arterial blood	9965	91.59425	24.91743
**003059_003**	O2SAT	Arterial blood	9906	97.02293	1.953542
**001014_002**	myelocytes	venous blood	9559	0.978345	0.667603
**001229_002**	Myelocyte count	venous blood	9430	0.074573	0.05779
**005440_001**	KL-6	Blood	9340	318.1546	162.6672
**005042_001**	Free Triiodothyronine	Blood	9259	2.659661	0.517869
**000032_001**	Iron	Blood	8703	69.39331	35.9601
**000034_001**	Total iron binding capacity	Blood	8053	300.8125	77.31101
**000033_001**	Unsaturated iron binding capacity	Blood	8030	224.3732	83.42148
**005018_007**	ferritin	Serum	8004	107.6098	108.4012
**005027_001**	CA15-3	Blood	7751	12.36051	6.786692
**005020_001**	C4	Blood	7514	21.29132	8.139746
**000021_001**	Cholinesterase	Blood	7477	296.2053	84.01567
**005019_001**	C3	Blood	7426	94.25909	22.87471
**001078_002**	Fibrinogen	venous blood	7251	433.5747	142.4854
**007403_007**	PSA	Serum	7244	1.582096	2.348575
**000041_001**	beta 2-microglobulin	Blood	6906	4.08523	2.77947
**000026_001**	Creatine kinase MB	Blood	6131	10.43141	5.372972
**002581_011**	Sodium	Fresh Urine	5636	77.01065	43.88488
**001230_002**	Post myelocyte count	venous blood	5447	0.05933	0.040833
**000008_001**	Direct bilirubin	Blood	5430	0.274733	0.193023
**001015_002**	Back myelocyte	venous blood	5255	0.688088	0.317499
**002583_011**	Potassium	Fresh Urine	5167	28.05284	16.06598
**000024_001**	Pancreatic amylase	Blood	5066	40.42696	19.66172
**005024_001**	CA19-9	Blood	4966	13.32716	9.990649
**001079_002**	Antithrombin III	venous blood	4631	79.81278	24.18135
**001023_002**	Atypical lymphocyte	venous blood	4094	0.905569	0.604413

**000070_001**	Rheumatoid factor	Blood	4026	27.34451	31.20005
**001231_002**	Atypical lymphocyte count	venous blood	3966	0.048432	0.032883
**005354_001**	Cardiac troponin T	Blood	3804	0.023936	0.026943
**011290_002**	O2SAT	venous blood	3687	68.87936	21.56587
**011289_002**	O2CT	venous blood	3684	12.25277	4.682811
**011295_002**	Hemoglobin	venous blood	3648	12.97525	2.505464
**011285_002**	Oxygen partial pressure	venous blood	3572	42.45677	15.5185
**011291_002**	Sodium	venous blood	3542	139.3001	3.496477
**011297_002**	FMetHb	venous blood	3539	0.838938	0.190485
**011292_002**	Potassium	venous blood	3537	3.840204	0.481714
**011284_002**	Carbon dioxide partial pressure	venous blood	3505	40.93498	7.029487
**011294_002**	Calcium ion	venous blood	3505	2.326117	0.104146
**011286_002**	HCO3	venous blood	3503	24.37568	3.188539
**002585_011**	Chlor	Fresh Urine	3491	72.53423	43.14348
**011300_002**	mOsm	venous blood	3485	286.3721	6.804761
**011293_002**	Chlor	venous blood	3469	106.7602	3.683814
**011288_002**	SBE	venous blood	3455	0.148452	3.153781
**000072_001**	IgA	Blood	3445	223.9419	117.3286
**011298_002**	Blood glucose	venous blood	3421	128.5177	34.65028
**011299_002**	Lactate	venous blood	3405	2.080969	1.147266
**005026_001**	CA125	Blood	3402	14.83665	9.349015
**011287_002**	Actual base excess	venous blood	3401	0.129403	2.890326
**011283_002**	pH	venous blood	3379	7.392305	0.0511
**011296_002**	COHb	venous blood	3346	1.02621	0.42742
**000073_001**	IgM	Blood	3303	89.90312	52.53756
**002626_011**	β2-microglobulin	Fresh Urine	3259	267.8868	347.283
**001081_002**	Fibrinogen Fibrin Degradation products	venous blood	3091	7.293303	6.77633
**005239_008**	intact PTH	Plasma	2907	129.7169	99.30982
**005516_001**	beta-D glucan	Blood	2792	8.027865	4.730145
**006962_007**	MMP-3	Serum	2784	64.32949	36.41494
**001012_002**	Erythroblasts	venous blood	2504	1.280751	0.5874
**005491_001**	Tacrolimus	Blood	2472	5.281837	3.152784
**002594_011**	Urea nitrogen	Fresh Urine	2469	468.7128	264.492
**005416_001**	Prealbumin	Blood	2465	19.90621	8.396696
**003003_001**	Blood glucose 60 min	Blood	2351	137.0336	29.38619
**000056_007**	HBs antibody	Serum	2263	0.655281	1.737646
**005279_007**	Estradiol	Serum	2064	43.17539	47.13189
**005037_007**	Follicle-stimulating hormone	Serum	2030	22.81754	24.10112
**002610_011**	Osmotic pressure	Fresh Urine	1916	436.6587	179.8706
**010014_007**	Docosahexaenoic acid	Serum	1860	124.1212	44.33893
**010011_007**	Dihomo-γ-linolenic acid	Serum	1850	34.44941	11.61128
**010012_007**	Arachidonic acid	Serum	1850	188.4154	47.94428

**002591_011**	Microalbumin	Fresh Urine	1835	26.57695	30.36117
**011196_007**	Highly sensitive HBs antigen	Serum	1811	0.107068	0.135332
**010013_007**	Eicosapentaenoic acid	Serum	1802	75.80472	43.06767
**010181_007**	EPA/AA ratio	Serum	1799	0.411679	0.242961
**005015_007**	HTLV-1 antibody	Serum	1765	0.101326	0.035452
**005276_001**	Glycoalbumin	Blood	1763	19.02479	4.172687
**007007_007**	HBc antibody (IgG)	Serum	1753	0.116885	0.043924
**005465_007**	Soluble IL-2 receptor	Serum	1746	452.8608	233.0241
**002592_011**	Microalbumin creatinine ratio	Fresh Urine	1713	31.25353	33.0308
**005023_001**	Alpha-fetoprotein	Blood	1711	3.552075	1.801064
**002581_012**	Sodium	urine collection	1694	77.89256	31.05785
**005630_007**	anti-SS-A/Ro antibody	Serum	1686	1.140095	0.366076
**002585_012**	Chlor	urine collection	1684	72.84382	28.93394
**002594_012**	Urea nitrogen	urine collection	1674	367.0066	156.4493
**002596_012**	creatinine	urine collection	1674	55.9307	23.20657
**002583_012**	Potassium	urine collection	1664	21.90144	9.758535
**005362_007**	Vitamin B12	Serum	1616	587.4697	308.0678
**002589_012**	Protein Determination	urine collection	1589	39.29264	42.44854
**002597_012**	creatinine 24 hr	urine collection	1536	949.1374	393.2739
**005350_008**	Human atrial natriuretic Peptide	Plasma	1535	62.57414	41.46055
**002595_012**	Urea Nitrogen 24 hours	urine collection	1511	6345.973	2747.185
**011152_001**	Procalcitonin	Blood	1497	0.24149	0.297556
**002584_012**	Potassium 24 hr	urine collection	1489	37.51242	17.36174
**000098_012**	Creatinine 24 hr	urine collection	1487	56.69469	22.16045
**002582_012**	Sodium 24 hours	urine collection	1484	133.6509	63.83039
**002586_012**	Chlor 24 hr	urine collection	1475	126.9763	61.74998
**004022_002**	Ammonia	venous blood	1457	47.52025	31.83074
**005626_007**	Anti-DNA antibody	Serum	1456	4.730769	3.710528
**005309_001**	cortisol	Blood	1401	9.793854	5.568887
**002598_012**	Uric acid	urine collection	1368	17.27924	10.23596
**002610_012**	Osmotic pressure	urine collection	1366	362.2057	112.8054
**002590_012**	Protein 24 hours	urine collection	1351	589.4878	642.8044
**005365_007**	Folic acid	Serum	1342	8.057004	4.215635
**000101_012**	Urine output 24 hours	urine collection	1337	1722.006	673.043
**000099_012**	Creatinine Clearance 24 hours	urine collection	1332	45.25946	30.29632
**005036_007**	Luteinizing hormone	Serum	1329	5.56757	5.034752
**000044_007**	Osmotic pressure	Serum	1323	281.5155	15.57487
**005054_007**	Total IgE	Serum	1313	190.2254	222.8283
**000093_001**	Vancomycin	Blood	1311	15.43217	5.288135
**005443_007**	SCC antigen	Serum	1299	1.141878	0.543587
**005878_007**	Rubella HI	Serum	1250	48.992	40.52863
**002599_012**	Uric Acid 24 hours	urine collection	1232	308.2687	183.1025

**004067_002**	Ethanol	venous blood	1174	12.92002	10.92349
**005455_007**	PIVKA-II	Serum	1109	21.80613	8.095734
**004071_004**	Hematocrit	umbilical cord blood	1074	48.17132	4.623018
**004068_004**	Sodium	umbilical cord blood	1073	138.4157	1.726283
**004078_004**	HCO3	umbilical cord blood	1072	22.97108	2.213469
**004079_004**	Actual base excess	umbilical cord blood	1068	-3.54588	2.31023
**004074_004**	hemoglobin	umbilical cord blood	1067	16.35904	1.542512
**004070_004**	Calcium ion	umbilical cord blood	1066	1.494775	0.063957
**004076_004**	Carbon dioxide partial pressure	umbilical cord blood	1066	47.91538	7.676305
**004073_004**	TCO2	umbilical cord blood	1062	24.44727	2.2971
**005265_007**	anti-thyroglobulin antibody	Serum	1054	2.300825	2.198458
**004077_004**	oxygen partial pressure	umbilical cord blood	1052	16.8384	4.714143
**011183_004**	Blood glucose level	umbilical cord blood	1048	77.37882	13.86001
**004075_004**	pH	umbilical cord blood	1047	7.294009	0.049483
**004080_004**	O2SAT	umbilical cord blood	1027	20.0224	9.628706
**004069_004**	Potassium	umbilical cord blood	1020	4.367647	0.342582
**007408_007**	Anti-CCP antibody	Serum	1017	0.563127	0.15159
**005459_007**	Cytokeratin 19 fragment	Serum	1000	1.5916	0.905776
**001025_002**	Hemogram Results 1	venous blood	992	17.54415	24.92287
**007240_007**	Cystatin C	Serum	982	1.454725	0.708554
**011251_007**	Concentration	Serum	968	0.516736	0.048652
**003005_001**	Blood glucose 120 min value	Blood	940	126.0202	25.04289
**002598_011**	Uric acid	Fresh Urine	938	30.40618	19.12857
**003001_001**	Blood glucose Before load	Blood	920	85.82283	8.107958
**001232_002**	Hemogram Item 1#	venous blood	894	0.564832	1.05188
**002600_011**	Calcium	Fresh Urine	893	8.531915	6.438423
**002005_020**	Fecal occult blood reaction 1-day method	stool	871	29.4845	58.56688
**005272_007**	anti-thyroid peroxidase antibody	Serum	814	1.41016	1.86804
**005233_008**	Adrenocorticotropic hormone	Plasma	782	18.04476	11.7354
**005444_007**	Neuron-specific enolase	Serum	777	10.70721	2.375937
**005464_008**	Gastrin-releasing peptide precursor	Plasma	748	58.4373	19.82295
**005261_007**	TSH receptor antibodies	Serum	741	1.498111	1.612316
**001212_002**	juvenile platelet ratio	venous blood	733	3.86794	2.578218
**001213_002**	Weak platelet count	venous blood	726	2.698898	2.107288
**005284_007**	Testosterone	Serum	725	2.310428	2.553581
**005282_007**	Progesterone	Serum	724	1.855663	3.228394
**001013_002**	Promyelocytes	venous blood	601	0.899168	0.652316
**005737_008**	Lupus anticoagulant	Plasma	598	1.119398	0.114267
**005472_001**	Cyclosporine	Blood	595	100.743	81.50975
**005354_007**	Cardiac troponin T	Serum	584	0.022286	0.022665
**005631_007**	anti-SS-B/La antibody	Serum	578	1.061592	0.2021
**010051_007**	Lung surfactant protein D	Serum	578	164.1166	111.7293

**005038_007**	prolactin	Serum	569	11.35694	6.458588
**001228_002**	Promyelocyte count	venous blood	564	0.078883	0.081142
**005235_007**	Somatomedin-C	Serum	563	133.1918	57.69245
**005424_007**	transferrin	Serum	557	271.6481	59.31466
**006144_019**	Urea breath test	exhaled breath	557	1.145242	2.13115
**005854_007**	Cytomegalovirus IgG EIA	Serum	527	16.35503	13.86452
**007111_007**	IgG subclass 4	Serum	511	92.7771	79.5628
**002602_011**	Inorganic phosphorus	Fresh Urine	504	34.94643	23.78338
**005095_007**	House dust 1	Serum	496	1.217742	1.511227
**005068_007**	Japanese cedar	Serum	490	1.969388	1.692363
**005427_007**	Zinc	Serum	482	61.97095	15.40848
**005609_007**	Serum complement titer	Serum	482	38.83693	10.34143
**005074_007**	Burn mite	Serum	481	1.328482	1.631781
**005313_008**	aldosterone	Plasma	479	129.2714	62.55432
**005071_007**	Hinoki (Hinoki cypress)	Serum	476	1.033613	1.235835
**005285_007**	Free testosterone	Serum	471	5.23121	3.641256
**002587_011**	Glucose	Fresh Urine	470	8.340426	8.662535
**011276_007**	Tartrate-resistant acid phosphatase	Serum	463	463.1404	229.1609
**010076_008**	HBV-DNA quantitative TaqMan	Plasma	452	2.329425	1.298745
**005361_008**	Renin activity	Plasma	444	0.995946	1.004429
**005405_007**	Angiotensin converting enzyme	Serum	444	15.51216	5.53755
**005497_007**	Helicobacter pylori Antibody	Serum	441	8.995465	10.61945
**005069_007**	Japanese cedar Concentration	Serum	439	5.040774	6.888529
**005262_007**	thyroglobulin	Serum	428	39.83508	41.10135
**005072_007**	Hinoki Concentration	Serum	420	0.73569	1.025736
**005096_007**	House dust 1 Concentration	Serum	419	0.99432	1.719768
**009060_001**	Positive Percentage	Blood	416	28.82476	9.985838
**009063_001**	CD8	Blood	414	878.4319	396.5499
**009062_001**	Positive Percentage	Blood	413	46.97845	9.881588
**009061_001**	CD4・CD8	Blood	411	521.7377	255.0613
**005075_007**	Burn mites Concentration	Serum	409	1.460073	2.658738
**009064_001**	CD4/CD8 ratio	Blood	397	0.620554	0.297514
**005367_001**	Vitamin B1	Blood	396	43.86414	14.06423
**005629_007**	anti-Sm antibody	Serum	383	1.072324	0.182095
**005456_007**	Sialyl Lex-i antigen	Serum	382	32.69764	7.080873
**005273_001**	Chorionic gonadotropin	Blood	377	157.5302	311.1366
**011154_007**	Free L-chain kappa chain	Serum	373	46.87721	50.48899
**005381_007**	Alkaline phosphatase isozyme 2	Serum	369	51.25745	17.6711
**005382_007**	Alkaline phosphatase isozyme 3	Serum	369	31.61789	19.72316
**005352_008**	Cerebral natriuretic peptide	Plasma	363	185.8077	205.2742
**005858_007**	Cytomegalovirus IgM EIA	Serum	353	0.554504	0.243535
**011155_007**	Free L-chain λ-chain	Serum	349	20.17825	15.70124

**011156_007**	Free L-chain kappa/λ ratio	Serum	349	1.671123	1.727639
**002633_041**	Glucose	spinal fluid	346	66.04335	13.97268
**005384_007**	Alkaline phosphatase isozyme 5	Serum	346	7.289017	8.526361
**005628_007**	anti-RNP antibody	Serum	346	2.034971	0.159409
**002616_011**	Albumin	Fresh Urine	344	56.73488	19.17203
**002615_011**	albumin-total globulin ratio	Fresh Urine	342	1.742544	1.100146
**011272_007**	Mac-2 binding protein glycosylated isomers	Serum	337	0.995341	0.643559
**002630_041**	Protein Determination	spinal fluid	330	52.96061	29.31085
**002618_011**	alpha 2 globulin	Fresh Urine	329	8.324924	4.841423
**002620_011**	gamma globulin	Fresh Urine	329	15.73982	7.627691
**005380_007**	Alkaline phosphatase isozyme 1	Serum	326	3.984663	5.332895
**005407_007**	Aldolase	Serum	326	4.971472	2.14693
**005408_007**	Lipase	Serum	325	45.25538	26.46205
**005861_007**	EB virus Anti-VCA IgG	Serum	324	87.53086	77.44417
**001545_040**	Erythroblast system rate	bone marrow	323	22.76254	11.75409
**001548_040**	myeloblast	bone marrow	323	9.594737	4.63766
**002619_011**	beta globulin	Fresh Urine	323	10.66068	3.315019
**005307_008**	homocysteine	Plasma	321	12.16822	4.034978
**011275_007**	Procollagen type 1-N- propeptide	Serum	320	51.37156	25.18078
**001542_040**	Polycytotic erythroblasts	bone marrow	319	11.68213	6.304268
**001551_040**	Lobular nuclear neutrophils	bone marrow	319	17.04702	8.192199
**005063_007**	ragweed Concentration	Serum	319	0.10373	0.01277
**001549_040**	Postmyelocyte	bone marrow	318	4.916667	2.281721
**001559_040**	Lymphocyte	bone marrow	318	13.06384	7.31721
**001550_040**	Rod-shaped nucleus neutrophils	bone marrow	317	9.290852	4.163055
**001543_040**	Orthochromatic erythroblast	bone marrow	316	9.447152	5.049088
**001558_040**	Myelomonocyte system rate	bone marrow	313	50.50703	14.22982
**005466_007**	Bone type Alkaline phosphatase	Serum	310	14.16452	5.53005
**002617_011**	Alpha 1 globulin	Fresh Urine	309	4.927508	2.152616
**002602_012**	Inorganic phosphorus	urine collection	307	30.11726	14.92024
**001547_040**	promyelocyte	bone marrow	306	2.77549	1.488895
**001561_040**	monocyte	bone marrow	302	3.576159	1.78979
**001541_040**	Basophilic erythroblasts	bone marrow	300	1.509333	1.266277
**005245_007**	C peptide	Serum	300	2.605667	1.685508
**011336_004**	HCO3	umbilical cord blood	296	24.13412	1.831241
**011340_004**	O2SAT	umbilical cord blood	295	29.69763	13.68348
**011343_004**	Chlor	umbilical cord blood	295	107.739	2.143573
**001535_040**	megakaryocytes	bone marrow	294	48.4483	51.55106
**011345_004**	Hemoglobin	umbilical cord blood	294	15.4449	1.421804
**011344_004**	Calcium ion	umbilical cord blood	293	2.845631	0.120587
**011338_004**	SBE	umbilical cord blood	292	-1.26918	1.826629
**011339_004**	O2CT	umbilical cord blood	292	6.210274	2.742167

**011350_004**	mOsm	umbilical cord blood	292	277.6288	3.775139
**001534_040**	Nucleated cells	bone marrow	290	101714.7	74291.23
**011334_004**	Carbon dioxide partial pressure	umbilical cord blood	290	50.01103	5.237516
**011335_004**	Oxygen partial pressure	umbilical cord blood	290	17.10517	4.300906
**011333_004**	pH	umbilical cord blood	289	7.30718	0.03727
**011341_004**	Sodium	umbilical cord blood	288	136.875	1.845514
**011337_004**	Actual base excess	umbilical cord blood	287	-2.2338	1.796463
**011346_004**	COHb	umbilical cord blood	286	0.091259	0.327286
**006512_007**	Mold Multi concentration	Serum	283	0.1	1.39E-17
**006981_008**	Amiodarone	Plasma	283	372.9028	200.7327
**011348_004**	Blood glucose	umbilical cord blood	282	70.15248	11.35428
**005669_011**	N-Acetylglucosaminidase	Fresh Urine	281	7.739858	7.013144
**001556_040**	acidophilic segmental nucleated neutrophil	bone marrow	280	1.296429	0.901167
**006980_008**	Amiodarone	Plasma	280	468.7625	251.0739
**011347_004**	FMetHb	umbilical cord blood	280	0.958571	0.160191
**011349_004**	Lactate	umbilical cord blood	278	1.98705	0.775513
**011342_004**	Potassium	umbilical cord blood	275	4.101818	0.288522
**005364_007**	25-OH vitamin D	Serum	271	16.21402	6.719327
**002603_012**	Inorganic phosphorus 24 hours	urine collection	269	567.8216	233.3717
**000090_001**	Valproic acid	Blood	265	43.52385	21.7963
**005845_007**	Varicella zoster IgG EIA	Serum	263	19.30342	13.88071
**002668_055**	potassium	peritoneal dialysis fluid	261	3.167816	0.75128
**002669_055**	Chlor	peritoneal dialysis fluid	261	99.56322	4.600254
**002692_055**	Glucose	peritoneal dialysis fluid	261	798.1648	391.538
**002672_055**	Urea nitrogen	peritoneal dialysis fluid	257	47.08755	13.8888
**002673_055**	creatinine	peritoneal dialysis fluid	256	6.587266	2.327275
**005321_008**	Adrenaline	Plasma	256	0.026016	0.018056
**002667_055**	Sodium	peritoneal dialysis fluid	254	130.4134	3.867159
**005322_008**	Noradrenaline	Plasma	253	0.268577	0.134006
**001560_040**	Plasma cells	bone marrow	250	1.0472	0.794669
**005077_007**	Porcelain mites	Serum	246	1.691057	1.856616
**001564_040**	Result 1	bone marrow	240	0.917917	0.695812
**005012_007**	HBe antibody	Serum	239	65.96109	39.94962
**000071_007**	IgG	Serum	231	1203.117	397.7865
**001553_040**	Acidophilic myelocyte	bone marrow	230	0.503478	0.307527
**002007_020**	Fecal occult blood reaction 2-day method	stool	230	14.68696	26.85376
**005060_007**	Chamogaya Concentration	Serum	228	0.10307	0.014305
**005867_007**	EB virus Anti-EBNA	Serum	228	26.53509	17.2009
**006994_007**	Anti-mitochondrial M2 antibody	Serum	223	1.519283	0.085611
**001555_040**	Acidophilic bacillary nucleus neutrophil	bone marrow	219	0.526027	0.342426
**002737_055**	protein	peritoneal dialysis fluid	217	75.65438	37.95789
**005428_007**	Copper	Serum	215	115.8744	26.79113

**002708_045**	lymphocyte	pleural fluid	214	44.76869	33.49576
**005501_007**	Toxoplasma IgM antibody	Serum	214	0.1	1.39E-17
**005620_007**	Anti-acetylcholine receptor binding antibody	Serum	211	1.934597	3.147575
**005078_007**	Solanum lycopersicum Concentration	Serum	210	3.079333	5.080285
**002707_045**	neutrophil	pleural fluid	209	25.4689	31.51629
**002670_045**	Total Protein	pleural fluid	207	3.395652	1.325197
**002671_045**	albumin	pleural fluid	205	1.799024	0.779202
**005636_007**	anti centromere antibody	Serum	205	2.313171	2.007601
**005836_007**	Herpes simplex IgG EIA	Serum	205	25.71756	25.84846
**005640_007**	anti-ds-DNAIgG antibody	Serum	204	5.17598	4.191907
**002535_012**	Specific gravity	urine collection	203	1.011901	0.004089
**002711_045**	Other blood cells	pleural fluid	203	29.99507	30.46195
**002654_041**	Nucleated cells	spinal fluid	202	5.960396	9.47888
**002713_045**	Specific gravity	pleural fluid	202	1.024946	0.007113
**003002_001**	Glucose 30 min	Blood	198	164.298	30.44688
**005009_007**	HBe antigen	Serum	198	0.314848	0.054444
**005044_007**	Insulin	Serum	198	12.02525	8.347891
**005098_007**	House dust 2	Serum	195	1.671795	1.751281
**005087_007**	Alternaria Alternaria concentration	Serum	194	0.1	1.39E-17
**011140_060**	Human papillomavirus genotype 1	fluid to be wiped out	194	42.79381	15.50426
**000091_001**	Digoxin	Blood	193	0.759223	0.374586
**002600_012**	Calcium	urine collection	191	4.486911	3.405659
**005188_007**	Insulin 60 min	Serum	191	43.27539	22.52159
**001554_040**	acidophilic postmyelocyte	bone marrow	190	0.427368	0.271559
**005187_007**	Insulin 30 min	Serum	189	34.97937	16.44642
**002679_045**	Lactate dehydrogenase	pleural fluid	188	212.4947	146.0965
**002851_045**	Nucleated cells	pleural fluid	187	848.0829	923.1021
**010081_007**	Anti-BP180 antibody	Serum	187	38.21444	46.12081
**005784_045**	Adenosine deaminase	pleural fluid	186	13.49839	7.154371
**005182_007**	Before insulin	Serum	185	5.063784	1.759643
**005190_007**	Insulin 120 min	Serum	184	32.21413	17.0257
**001557_040**	basophil	bone marrow	183	0.328962	0.139002
**002692_045**	Glucose	pleural fluid	182	120.456	27.71253
**002587_012**	Glucose	urine collection	181	13.05525	18.37321
**002714_045**	pH	pleural fluid	180	7.104444	0.145634
**005259_007**	TSH-stimulating receptor antibodies	Serum	179	109.6927	20.50241
**005458_007**	DUPAN-2	Serum	178	28.41573	8.53879
**000156_001**	Pediatric eGFR Calculated value	Blood	177	108.5141	27.06342
**005017_007**	anti-streptolysin-O antibody	Serum	176	97.46023	109.2485
**002676_045**	Cholesterol	pleural fluid	175	57.97714	28.63925
**005346_007**	Erythropoietin	Serum	172	21.60814	20.41479
**005535_007**	Chlamydia trachomatis IgA & IgG	Serum	172	0.191703	0.196727

**005409_007**	Lysozyme	Serum	170	7.518235	2.807148
**005880_007**	Rubella IgG EIA	Serum	170	15.64412	13.45515
**005840_007**	Herpes simplex IgM EIA	Serum	169	0.224083	0.120052
**005099_007**	House dust 2 Concentration	Serum	168	2.926667	4.826208
**005537_007**	Chlamydia trachomatis IgA&IgG	Serum	168	0.116744	0.129905
**005849_007**	Varicella zoster IgM EIA	Serum	166	0.254819	0.075162
**002677_045**	Neutral Fat	pleural fluid	164	16.39024	10.01196
**002686_046**	Pancreatic amylase	ascites	161	612.8385	1018.241
**005019_007**	C3	Serum	160	95.96875	22.69913
**002601_012**	Calcium 24 hours	urine collection	159	84.03145	56.28357
**007015_011**	Collagen cross-linked N-terpeptide type 1 Telopeptide	Fresh Urine	159	37.0327	22.80685
**005020_007**	C4	Serum	157	22.36943	7.945557
**005741_008**	Protein C activity	Plasma	155	112.5226	26.01856
**000072_007**	IgA	Serum	152	220.4408	102.1366
**000073_007**	IgM	Serum	151	98.11921	47.73244
**005872_007**	Measles IgG EIA	Serum	151	16.25033	11.55349
**005040_007**	Thyroxine	Serum	149	6.590805	1.25378
**001562_040**	Reticulocytes	bone marrow	147	0.273469	0.124045
**002664_041**	Specific gravity	spinal fluid	142	1.005627	0.000648
**005731_008**	Thrombin-antithrombin complex	Plasma	142	2.728169	2.413272
**005785_045**	Hyaluronic acid	pleural fluid	138	18.06522	12.95089
**010120_007**	TARC	Serum	136	539.7059	414.0374
**002666_041**	pH	spinal fluid	134	7.98209	0.226729
**002765_015**	Semen volume	semen	133	5.025564	1.46534
**002757_015**	Sperm motility	semen	131	50.74275	19.66171
**002755_015**	Sperm concentration	semen	130	52.909	37.35258
**002767_015**	Semen pH	semen	127	8.114961	0.179098
**002777_015**	Abstinence period	semen	127	4.110236	2.193889
**005234_008**	Antidiuretic hormone	Plasma	127	2.014961	1.315842
**005363_007**	1.25-(OH)2 vitamin D	Serum	127	48.03937	32.73545
**005975_007**	Human parvo B19 IgM	Serum	124	0.428387	0.205827
**005884_007**	Rubella IgM EIA	Serum	123	0.213171	0.098583
**003004_001**	Blood glucose 90 min	Blood	122	142.5	40.88671
**005442_007**	NCC-ST-439	Serum	122	3.159016	3.330345
**011256_007**	Anti-glomerular basement membrane antibody	Serum	122	0.642623	0.264393
**000089_001**	Carbamazepine	Blood	121	5.750992	2.447949
**002588_012**	Glucose 24 hr	urine collection	121	588.3223	749.2754
**005750_008**	Protein S (free antigen level)	Plasma	119	81.37815	22.5381
**007010_001**	Cytomegaloantigen	Blood	118	8.135593	12.73592
**001538_040**	Basophilic megakaryoblasts	bone marrow	117	0.28547	0.126808

**005262_001**	Thyroglobulin	Blood	117	55.48154	61.59612
**005315_007**	Dehydroepiandrosterone sulfate	Serum	117	115.0427	69.37261
**005089_007**	Feline debris	Serum	116	0.336207	0.709488
**005189_007**	Insulin 90 min	Serum	116	34.54569	17.88406
**005972_007**	Human parvo B19 IgG	Serum	116	6.453707	4.375223
**005232_007**	Growth hormone	Serum	114	0.648596	0.616296
**005891_007**	Mumps IgG EIA	Serum	113	4.390265	2.140384
**005039_007**	Triiodothyronine	Serum	110	84.71818	15.17913
**011234_010**	Methanephrine Concentration	urine	110	0.107818	0.064742
**011174_001**	Prothrombin time	Blood	107	304.9439	29.23452
**011235_010**	normetanephrine concentration	urine	106	0.230377	0.127451
**005090_007**	Feline debris Concentration	Serum	105	0.179048	0.208523
**011172_001**	Prothrombin International Normalized Ratio	Blood	105	1.977143	0.659145
**005432_001**	Platelet surface IgG	Blood	103	43.75922	25.7503
**005093_007**	Canine debris Concentration	Serum	102	0.10549	0.020996
**010119_007**	Anti-Mullerian Tube	Serum	102	2.267255	2.160513
**005355_007**	Hyaluronic acid	Serum	101	48.43663	32.62822
**002675_046**	Total bilirubin	ascites	100	0.718	0.527311
**006966_007**	Type 1 collagen cross-linked N- telopeptide	Serum	98	17.88878	5.252155
**010070_007**	HCV-RNA quantitative TaqMan	Serum	98	5.859184	1.045643
**006503_007**	Rice mulch Concentration	Serum	96	0.116563	0.049754
**006724_007**	Anisakis - class	Serum	89	1.730337	1.917409
**005400_007**	Lactate dehydrogenase 1	Serum	87	21.90805	10.15191
**005402_007**	Lactate dehydrogenase 3	Serum	87	21.56322	6.344199
**000088_001**	Phenytoin	Blood	85	8.128706	5.240659
**005047_007**	Tobramycin	Serum	84	9.165476	5.698668
**005446_007**	Elastase 1	Serum	84	88.13095	16.60511
**002851_055**	Nucleated cells	peritoneal dialysis fluid	83	56.1506	78.8873
**002616_012**	albumin	urine collection	82	54.10366	20.32412
**011176_007**	Voriconazole	Serum	82	2.145244	1.551541
**002615_012**	albumin-total globulin ratio	urine collection	81	1.620247	1.17725
**005401_007**	Lactate dehydrogenase 2	Serum	81	34.12346	6.05265
**005404_007**	Lactate dehydrogenase 5	Serum	81	8.740741	5.571754
**005421_007**	haptoglobin 2-2	Serum	81	75.1358	47.14864
**002604_011**	Magnesium	Fresh Urine	80	7.175	5.369334
**002618_012**	alpha 2 globulin	urine collection	80	10.2675	6.960299
**002656_041**	Mononuclear cell count	spinal fluid	80	35.3	33.19654
**005403_007**	Lactate dehydrogenase 4	Serum	80	9.35	3.368131
**006725_007**	Anisakis (genus) Concentration	Serum	79	6.402405	11.62804
**002617_012**	Alpha 1 globulin	urine collection	77	6.538961	2.885543
**002709_045**	Eosinophils	pleural fluid	77	3.525974	4.396392
**002619_012**	beta globulin	urine collection	76	11.09079	4.049401

**002620_012**	gamma globulin	urine collection	76	15.61447	8.281654
**002671_046**	albumin	ascites	76	1.178947	0.880502
**006365_007**	Pine (genus) Concentration	Serum	76	0.1	2.79E-17
**005360_007**	Tyrosine	Serum	75	98.64	30.34649
**001544_040**	Mitosis of erythroblast nuclei	bone marrow	74	0.2	5.59E-17
**005357_007**	Total branched chain amino acid/tyrosine molar ratio	Serum	73	4.763973	1.97235
**005740_008**	Protein C	Plasma	72	105.25	23.24017
**005503_007**	Anti-streptokinase antibody	Serum	71	593.8028	741.3547
**002732_048**	Lymphocytes	joint fluid	69	6.978261	6.886386
**005594_007**	Hepatitis A antibody	Serum	68	0.121176	0.032896
**005359_007**	branched chain amino acid	Serum	67	414.209	93.77997
**007015_010**	Type 1 collagen cross-linked N- Telopeptide	urine	66	24.27273	14.2456
**002854_048**	monocyte	joint fluid	65	5.138462	3.883567
**002655_041**	Polynuclear cell count	spinal fluid	64	6.890625	11.16843
**005120_007**	Wheat Concentration	Serum	64	0.1	1.40E-17
**005441_007**	Serum amyloid A protein	Serum	64	29.17344	40.1853
**001539_040**	Polycytotic megakaryoblasts	bone marrow	63	0.257143	0.110299
**002731_048**	Neutrophils	joint fluid	63	87.62698	9.571871
**002713_046**	Specific gravity	ascites	62	1.018645	0.008144
**011273_007**	Collagen type 4S	Serum	62	5.316129	1.628455
**005420_007**	haptoglobin 2-1	Serum	61	99.60656	64.32943
**005713_011**	Alpha 1 microglobulin	Fresh Urine	61	18.46393	16.24868
**002670_046**	Total Protein	ascites	60	2.068333	1.584083
**002708_046**	lymphocyte	ascites	60	29.59167	21.51477
**005066_007**	Wormwood Concentration	Serum	60	0.1	4.20E-17
**011220_048**	Nucleated cells	joint fluid	60	10751.88	12102.23
**002853_045**	monocyte	pleural fluid	59	1.389831	0.914763
**004068_022**	Sodium	venous blood	59	139.5593	1.896196
**004076_022**	Carbon dioxide partial pressure	venous blood	59	50.59661	6.697065
**005274_007**	Chorionic gonadotropin beta subunit	Serum	59	0.1	2.80E-17
**006394_007**	Moth class	Serum	59	0.389831	0.743175
**011263_007**	Anti-MDA5 antibody	Serum	59	4.525424	0.50364
**002711_046**	Other blood cells	ascites	58	48.22414	27.84515
**005244_008**	Parathyroid hormone-related protein	Plasma	58	1.1	2.24E-16
**002679_046**	Lactate dehydrogenase	ascites	57	120.9649	107.5936
**004070_022**	Calcium ion	venous blood	57	1.508596	0.066183
**004073_022**	TCO2	venous blood	57	26.33333	1.835497
**004075_022**	pH	venous blood	57	7.303947	0.033583
**004078_022**	HCO3	venous blood	57	24.79123	1.700555
**011183_022**	Blood glucose level	venous blood	57	62.63158	8.446537
**001029_002**	Hemogram 3	venous blood	56	1.267857	0.587478

**002851_046**	Nucleated cells	ascites	56	251.8304	337.5384
**004069_022**	Potassium	venous blood	56	4.178571	0.398047
**004071_022**	Hematocrit	venous blood	56	44.19643	4.408338
**004074_022**	hemoglobin	venous blood	56	15.02321	1.494717
**004077_022**	Oxygen partial pressure	venous blood	56	16.57143	4.022017
**004079_022**	Actual base excess	venous blood	56	-1.53571	1.628879
**010140_007**	Pertussis toxin	Serum	56	24.57143	20.40486
**010141_007**	pertussis fibrous erythrocyte aggregate	Serum	56	25.25	22.2394
**002692_046**	Glucose	ascites	55	120.4	41.67093
**005463_007**	Type I collagen C-terminal telopeptide	Serum	55	5.74	1.903953
**004080_022**	O2SAT	venous blood	54	19.05556	7.431236
**011231_010**	Noradrenaline	urine	54	231.6648	137.1851
**002707_046**	neutrophil	ascites	53	13.5283	15.96222
**005113_007**	Shrimp	Serum	53	0.358491	0.709668
**006395_007**	Moth Concentration	Serum	52	0.183077	0.183105
**002710_045**	basophil	pleural fluid	51	1.019608	0.640006
**005768_041**	Adenosine deaminase	spinal fluid	51	1.62549	1.117648
**011230_010**	Adrenaline	urine	51	15.89608	9.539182
**002606_011**	Amylase	Fresh Urine	50	212.62	160.2112
**010112_008**	Aldosterone Before loading	Plasma	50	155.6	75.95622
**011232_010**	dopamine	urine	50	791.6	445.6427
**006178_008**	Renin Activity Before	Plasma	49	0.463265	0.318652
**002535_013**	Specific gravity	early morning urine	48	1.017958	0.008344
**011138_007**	Hepatitis B virus core-associated antigen	Serum	48	3.033333	0.078098
**002541_013**	pH	early morning urine	47	5.968085	0.754726
**005114_007**	Shrimp concentration	Serum	47	0.175957	0.168583
**005749_008**	Protein S activity	Plasma	47	78.70213	29.61779
**006404_007**	Cockroaches Concentration	Serum	47	0.102553	0.007931
**007205_008**	Factor VIII activity	Plasma	47	94.59574	48.80024
**002714_046**	pH	ascites	46	7.091304	0.182362
**006803_007**	Gluten Concentration	Serum	45	0.1	4.21E-17
**005752_008**	von Willebrand Factor activity	Plasma	44	147.3409	77.76409
**007209_007**	Anti-desmoglein 1 antibody	Serum	44	14.05909	21.69611
**011227_001**	CD19	Blood	44	1.888636	2.39488
**005240_007**	calcitonin	Serum	43	1.366047	1.167382
**005392_007**	Creatine kinase MM	Serum	43	97.06977	1.36966
**010160_007**	omega-5 gliadin concentration	Serum	43	0.1	2.81E-17
**002589_013**	Protein determination	early morning urine	42	12.2619	10.6635
**002667_046**	Sodium	ascites	42	139.5714	4.434581
**002677_046**	Neutral Fat	ascites	42	49.71429	40.52779
**005143_008**	Factor XII activity	Plasma	42	83.69048	34.43586
**005742_008**	Protein S	Plasma	42	89.28571	12.9413

**002596_013**	creatinine	early morning urine	41	109.8049	73.84044
**002668_046**	Potassium	ascites	41	4.336585	0.516602
**005247_007**	Gastrin	Serum	41	1121.512	1225.259
**005391_007**	Creatine kinase MB	Serum	41	2.292683	0.873038
**006689_007**	Goose feathers - Concentration	Serum	41	0.1	1.41E-17
**011141_060**	Human papillomavirus genotype 2	fluid to be wiped out	41	53.90244	3.176514
**002735_048**	Other blood cells	joint fluid	40	2.65	2.267948
**005057_007**	Hargaya Concentration	Serum	40	0.1275	0.060373
**005111_007**	Crab Concentration	Serum	39	0.103077	0.012387
**005370_007**	Pyridoxamine	Serum	39	0.2	2.81E-17
**011128_007**	Trichosporon asahi Antibody	Serum	38	0.032105	0.052048
**011445_007**	Chlamydia pneumoniae IgG	Serum	38	48.60526	35.08025
**005471_007**	Zonisamide	Serum	37	18.28378	10.38379
**005875_007**	Measles IgM EIA	Serum	37	0.119189	0.051606
**010170_007**	Pro-collagen III peptide	Serum	37	0.678378	0.170188
**004103_041**	Polymorphonuclear cell ratio	spinal fluid	36	39.60278	33.82817
**004104_041**	Mononuclear cell ratio	spinal fluid	36	57.05	32.91382
**006359_007**	Acinonychium sp. concentration	Serum	36	0.1	1.41E-17
**006613_007**	Alder (genus)-class	Serum	36	0.333333	0.717137
**010115_008**	Aldosterone 60 min value	Plasma	36	117.5	53.48938
**011442_007**	Chlamydia pneumoniae IgA	Serum	36	4.694444	3.912029
**002629_041**	Chlor	spinal fluid	35	125.0286	4.025549
**002707_055**	neutrophil	peritoneal dialysis fluid	35	73.37143	35.4808
**004102_041**	Mononuclear cell count	spinal fluid	35	96.77143	75.08626
**010116_008**	Aldosterone 90 min value	Plasma	35	111.8	47.16417
**002672_046**	Urea nitrogen	ascites	34	17.35	7.63196
**002673_046**	creatinine	ascites	34	0.933235	0.408187
**005588_007**	Hepatitis A antibody	Serum	34	0.225	0.106351
**005738_008**	Plasminogen	Plasma	34	87.73529	23.29431
**006181_008**	Renin activity 60 min value	Plasma	34	0.482353	0.345947
**006182_008**	Renin activity 90 min value	Plasma	34	0.455882	0.31449
**007210_007**	Anti-Desmoglein 3 antibody	Serum	34	16.88824	21.91986
**005371_007**	Pyridoxal	Serum	33	5.324242	3.480933
**005423_007**	ceruloplasmin	Serum	33	21.76364	6.910717
**006614_007**	Alder (genus) Concentration	Serum	33	0.176061	0.183387
**004101_041**	Polynuclear cell count	spinal fluid	32	84.09375	122.1106
**005289_007**	Apolipoprotein B	Serum	32	99.1875	19.93609
**006616_007**	Birch (genus) -class	Serum	32	0.9375	1.522678
**011315_009**	O2SAT	hematochezia	32	73.8	13.10986
**011320_009**	Hemoglobin	hematochezia	32	10.51875	1.901177
**011322_009**	FMetHb	hematochezia	32	0.953125	0.30052
**011326_009**	FiO2	hematochezia	32	43.51875	23.06103

**001552_040**	Acidophilic promyelocyte	bone marrow	31	0.277419	0.111683
**002669_046**	Chlor	ascites	31	107.8065	5.600115
**004106_041**	Nucleated cells	spinal fluid	31	172.2258	152.6446
**004108_041**	Other cells	spinal fluid	31	1.322581	1.661066
**005084_007**	Candida concentration	Serum	31	0.11871	0.046097
**005498_007**	Cold agglutination reaction	Serum	31	2248.129	3575.434
**006460_007**	Apples - class	Serum	31	0.83871	1.240881
**006469_007**	Peach - Class	Serum	31	0.935484	1.23654
**011316_009**	Sodium	hematochezia	31	138.1935	3.876591
**011321_009**	COHb	hematochezia	31	1.196774	0.338116
**011324_009**	Lactate	hematochezia	31	2.774194	2.50346
**011325_009**	mOsm	hematochezia	31	284.8129	8.131984
**000087_001**	Phenobarbital	Blood	30	16.69467	5.398762
**000092_001**	Theophylline	Blood	30	6.236667	4.527186
**002711_085**	Other blood cells	Other	30	56.98333	31.54102
**004109_041**	Other cell ratio	spinal fluid	30	0.706667	1.055995
**005102_007**	Egg white Concentration	Serum	30	0.108667	0.027635
**006463_007**	Kiwi - Class	Serum	30	0.5	0.820008
**006476_007**	Mackerel Concentration	Serum	30	0.1	2.82E-17
**011314_009**	O2CT	hematochezia	30	10.31333	2.518721
**011318_009**	Chlor	hematochezia	30	108.9	3.220088
**011319_009**	Calcium ion	hematochezia	30	2.178333	0.171506
**011323_009**	Blood glucose	hematochezia	30	140.8333	49.1641
**001540_040**	Orthochromatic megakaryoblasts	bone marrow	29	0.248276	0.102193
**011312_009**	Actual base excess	hematochezia	29	-3.64828	4.65285
**011313_009**	SBE	hematochezia	29	-3.54828	4.950009
**011317_009**	Potassium	hematochezia	29	3.934483	0.545289
**002851_085**	nucleated cell	Other	28	430.1786	307.1064
**005105_007**	Egg yolk Concentration	Serum	28	0.1	1.41E-17
**007022_008**	Interleukin-6	Plasma	28	15.98821	11.85924
**011308_009**	pH	hematochezia	28	7.352036	0.066005
**011311_009**	HCO3	hematochezia	28	21.99643	3.7025
**001536_040**	Proerythroblasts	bone marrow	27	0.259259	0.108342
**005470_007**	Clonazepam	Serum	27	18.32222	11.1393
**005803_056**	CD4	Bronchoalveolar Lavage Fluid	27	52.2037	16.87416
**006461_007**	Apples - Class	Serum	27	0.568148	0.906201
**006464_007**	Kiwi - Concentration	Serum	27	0.263704	0.323147
**006509_007**	Animal epithelium Mulch concentration	Serum	27	0.120741	0.06656
**011307_009**	Body temperature	hematochezia	27	36.86667	0.428773
**002708_085**	lymphocyte	Other	26	11.38462	9.331996
**002711_055**	Other blood cells	peritoneal dialysis fluid	26	2.346154	1.853479
**005804_056**	CD8	Bronchoalveolar Lavage Fluid	26	22.68462	9.086152

**006470_007**	Peach - Class	Serum	26	0.569615	0.82391
**011309_009**	Carbon dioxide partial pressure	hematochezia	26	41.4	4.833549
**011310_009**	Oxygen partial pressure	hematochezia	26	39.22308	5.694721
**002708_055**	lymphocyte	peritoneal dialysis fluid	25	4.38	4.682147
**006186_007**	Cortisol Before	Serum	25	8.842	4.858138
**006506_007**	Weed mulch Concentration	Serum	25	0.1	1.42E-17
**009121_056**	CD4/CD8 ratio	Bronchoalveolar Lavage Fluid	25	2.4056	1.290294
**010105_007**	Inulin blood concentration (2)	Serum	25	17.716	4.94315
**003029_001**	ICG stagnation rate 15 min value	Blood	24	15.91667	8.605694
**005108_007**	Milk Concentration	Serum	24	0.1	1.42E-17
**005142_008**	Factor XI activity	Plasma	24	76.45833	26.8425
**005241_007**	Osteocalcin	Serum	24	14.09167	8.960343
**005664_011**	Creatinine Corrected value	Fresh Urine	24	188.8125	163.5502
**006160_008**	Before adrenocorticotropic hormone	Plasma	24	20.29583	15.44061
**006617_007**	Birch (genus) Concentration	Serum	24	0.145417	0.139096
**010104_007**	Inulin blood concentration (1)	Serum	24	16.39167	3.165564
**010106_012**	Inulin Urinary Concentration	urine collection	24	256.7917	148.287
**002604_012**	magnesium	urine collection	23	6.304348	3.363369
**002627_041**	Sodium	spinal fluid	23	148.2174	3.919143
**002707_085**	neutrophil	Other	23	10.63043	14.15356
**005663_011**	Creatinine	Fresh Urine	23	88.98043	51.70233
**006188_007**	Cortisol 30 min value	Serum	23	17.87522	4.002538
**006466_007**	Melon - Class	Serum	23	0.130435	0.34435
**006467_007**	Melon - Class	Serum	23	0.161739	0.121754
**002676_046**	Cholesterol	ascites	22	26.13636	13.33201
**002685_046**	Amylase	ascites	22	30.27273	12.69114
**002853_046**	monocyte	ascites	22	1.681818	1.401916
**005160_011**	Fibrin/fibrinogen degradation products	Fresh Urine	22	29	30.09157
**005662_011**	Transferrin	Fresh Urine	22	12384.4	11884.39
**006189_007**	Cortisol 60 min value	Serum	22	19.66318	3.364281
**010078_008**	ADAMST13 activity	Plasma	22	0.703636	0.243966
**010108_012**	Inulin Urinary Concentration Urine Volume 60 min	urine collection	22	281.1364	153.6069
**010109_012**	Inulin clearance	urine collection	22	58.72727	36.50725
**011230_011**	Adrenaline	Fresh Urine	22	17.35455	11.16072
**001568_040**	Bone marrow puncture	bone marrow	21	0.2	2.84E-17
**002686_045**	Pancreatic amylase	pleural fluid	21	38.47619	38.43256
**005748_008**	alpha 2 Plasmin inhibitor plasmin complex	Plasma	21	1.519048	1.153525
**005894_007**	Mumps IgM EIA	Serum	21	0.129048	0.075426
**011231_011**	Noradrenaline	Fresh Urine	21	185.2905	121.899
**002628_041**	Potassium	spinal fluid	20	2.89	0.161897
**005144_008**	Factor XIII quantification	Plasma	20	90.25	27.71068

**005337_007**	Acetoacetic acid	Serum	20	1541.1	1852.289
**005338_007**	3-Hydroxybutyric acid	Serum	20	4487.95	6036.079
**005339_007**	Total ketone bodies	Serum	20	6029.05	7751.966
**005832_007**	Herpes simplex CF	Serum	20	7.2	5.287523
**006341_007**	Concentration of O. aureus	Serum	20	0.1585	0.1339
**011232_011**	dopamine	Fresh Urine	20	705.5	475.5216
**011238_011**	Vanillylmandelic acid	Fresh Urine	20	3.585	1.1762
**011239_011**	Vanillylmandelic acid concentration	Fresh Urine	20	3.685	2.263736
**011240_011**	creatinine concentration	Fresh Urine	20	113.461	74.9014
**002605_012**	Magnesium 24 hours	urine collection	19	129.6316	72.09347
**002693_045**	Osmotic pressure	pleural fluid	19	289.0526	9.17408
**005140_008**	Factor IX activity	Plasma	19	98	20.27588
**005688_012**	Metanephrine Daily Dose	urine collection	19	0.135263	0.058913
**006187_007**	Cortisol 15 min value	Serum	19	15.96263	3.14121
**006436_007**	Peanuts - Class	Serum	19	0.368421	0.760886
**011253_012**	Methanephrine Concentration	urine collection	19	0.088421	0.05429
**005654_012**	C peptide	urine collection	18	36.52222	42.5485
**005689_012**	daily dose of normetanephrine	urine collection	18	0.247222	0.094483
**005833_007**	Herpes simplex type 1 NT	Serum	18	7.111111	5.05137
**011252_012**	Methanephrine Total Daily Dose	urine collection	18	0.376667	0.129706
**001004_045**	hematocrit	pleural fluid	17	0.070588	0.11048
**002685_045**	Amylase	pleural fluid	17	32.64706	16.80008
**005473_007**	Lithium carbonate	Serum	17	0.623529	0.317272
**011235_011**	Normetanephrine Concentration	Fresh Urine	17	0.255294	0.160005
**011254_012**	Normetanephrine Concentration	urine collection	17	0.147059	0.075064
**001546_040**	Myeloblasts	bone marrow	16	0.2875	0.10247
**005597_007**	IgM-HBc antibody	Serum	16	0.11125	0.055
**005852_007**	Cytomegalo CF	Serum	16	8.75	8.160882
**005937_007**	Coxsackie B3 Type NT	Serum	16	22.25	23.13871
**005939_007**	Coxsackie Type B4 NT	Serum	16	5.25	1.914854
**006437_007**	Peanuts - class	Serum	16	0.135625	0.085086
**006515_007**	Food mulch concentration	Serum	16	0.101875	0.0075
**002675_045**	Total bilirubin	pleural fluid	15	1.286667	1.454484
**005305_007**	lipoprotein	Serum	15	14.55333	9.461642
**005329_061**	Lactic acid	Protein removal	15	11.64	2.06079
**005493_007**	Clobazam	Serum	15	78.42	64.67899
**005717_012**	Noradrenaline	urine collection	15	144.9067	49.80801
**005746_008**	C1-inactivator activity	Plasma	15	108.2	18.68995
**005941_007**	Coxsackie Model B5 NT	Serum	15	4.8	1.656157
**006472_007**	Bananas - Class	Serum	15	0.4	0.736788
**006473_007**	Bananas - Concentration	Serum	15	0.246667	0.245027
**010142_008**	Anti-HIT antibody	Plasma	15	0.6	1.15E-16

**011234_011**	Methanephrine concentration	Fresh Urine	15	0.09	0.04504
**001001_045**	White Blood Cell Count	pleural fluid	14	1.428571	0.931028
**002669_045**	Chlor	pleural fluid	14	107.9286	8.57065
**005255_007**	Insulin antibodies	Serum	14	0.4	5.76E-17
**005419_007**	Haptoglobin 1-1	Serum	14	132.6429	61.1802
**005494_007**	Desmethylclobazam	Serum	14	342.8571	212.3494
**005573_007**	HCV serotypes Grouping	Serum	14	1.285714	0.468807
**005716_012**	Adrenaline	urine collection	14	8.821429	5.236437
**005718_012**	Dopamine	urine collection	14	880.7143	421.3251
**005932_007**	Coxsackie Model A16 NT	Serum	14	5.142857	1.875229
**006422_007**	Cheese concentration	Serum	14	0.1	1.44E-17
**006482_007**	Sardines Concentration	Serum	14	0.1	1.44E-17
**007352_007**	Oysters Concentration	Serum	14	0.1	1.44E-17
**011243_011**	homovanillic acid	Fresh Urine	14	4.464286	2.229732
**011244_011**	homovanillic acid concentration	Fresh Urine	14	4.064286	2.698036
**011245_011**	creatinine concentration	Fresh Urine	14	109.26	75.93954
**001002_045**	Red Blood Cell Count	pleural fluid	13	0.006923	0.009473
**002668_045**	Potassium	pleural fluid	13	4.115385	0.57278
**002709_085**	eosinophil	Other	13	1.653846	1.546294
**002853_055**	monocyte	peritoneal dialysis fluid	13	43.57692	29.74534
**005172_010**	Fibrin/fibrinogen degradation products	urine	13	146	74.71724
**005368_008**	Vitamin B2	Plasma	13	3.269231	0.834051
**005669_010**	N-Acetylglucosaminidase	urine	13	3.030769	2.327869
**005933_007**	Coxsackie Model B1 NT	Serum	13	5.230769	3.419402
**005935_007**	Coxsackie Model B2 NT	Serum	13	10.15385	8.101915
**006183_008**	Renin activity	Plasma	13	3.130769	2.980879
**006428_007**	Barley concentration	Serum	13	0.1	1.44E-17
**006479_007**	Horse mackerel Concentration	Serum	13	0.1	1.44E-17
**010117_008**	Aldosterone	Plasma	13	344.3846	227.0934
**002693_046**	Osmotic pressure	ascites	12	296.5833	23.86499
**002709_046**	Eosinophils	ascites	12	0.666667	0.325669
**005117_007**	Tuna concentration	Serum	12	0.1	1.45E-17
**005123_007**	Rice Concentration	Serum	12	0.1	1.45E-17
**005308_007**	High-sensitivity CRP	Serum	12	0.01825	0.01572
**005373_007**	Vitamin C	Serum	12	7.816667	5.835601
**005447_007**	Tissue polypeptide antigen	Serum	12	16	10.8962
**006410_007**	Cladosporium concentration	Serum	12	0.1	1.45E-17
**006439_007**	Sesame - Class	Serum	12	1.083333	1.311372
**006797_007**	Latex Concentration	Serum	12	0.124167	0.051779
**006835_007**	Almonds - Class	Serum	12	0.833333	1.114641
**007222_007**	HER2 protein quantification	Serum	12	14.89167	1.111476
**007361_007**	Scallops	Serum	12	0.083333	0.288675

**007362_007**	Scallops Concentration	Serum	12	0.160833	0.152044
**001570_040**	Sideroblast type 1	bone marrow	11	18.54545	10.65236
**001571_040**	Sideroblast type 2	bone marrow	11	13	8.024961
**001572_040**	Sideroblast type 3	bone marrow	11	4.636364	4.410731
**002667_045**	Sodium	pleural fluid	11	142.6364	2.873072
**002670_047**	Total Protein	Pericardial fluid	11	4.445455	1.165645
**002692_047**	Glucose	Pericardial fluid	11	105.7273	73.17116
**002710_046**	basophil	ascites	11	0.590909	0.20226
**005347_007**	Myoglobin	Serum	11	327.1636	259.6443
**005468_007**	L3 fraction	Serum	11	40.89091	34.85678
**005914_007**	Influenza A CF	Serum	11	10.90909	5.957425
**005965_007**	Echo type 22 NT	Serum	11	9.454545	4.48026
**006240_007**	Methotrexate 48 hour value	Serum	11	0.176727	0.092019
**006241_007**	Methotrexate 72 hour value	Serum	11	0.058727	0.023605
**006401_007**	Chironomidae (genus) Concentration	Serum	11	0.160909	0.148355
**006836_007**	Almonds-Concentration	Serum	11	0.386364	0.404581
**010126_007**	L1 fraction	Serum	11	59.14545	34.90313
**011142_060**	Human papillomavirus genotype 3	fluid to be wiped out	11	57.36364	1.120065
**011279_008**	Presepsin	Plasma	11	494.8182	309.828
**002671_047**	albumin	Pericardial fluid	10	2.25	0.494975
**002679_047**	Lactate dehydrogenase	Pericardial fluid	10	1614.6	2316.625
**002707_047**	neutrophil	Pericardial fluid	10	61.1	35.70699
**002713_047**	Specific gravity	Pericardial fluid	10	1.0311	0.006208
**005173_041**	Fibrin/fibrinogen degradation products	spinal fluid	10	2.72	0.818942
**005304_007**	remnant-like lipoprotein cholesterol titero	Serum	10	6.03	3.488409
**005330_061**	Pyruvic acid	Protein removal	10	0.68	0.193218
**005843_007**	Varicella zoster CF	Serum	10	7.6	5.796551
**005925_007**	Coxsackie A4 NT	Serum	10	18.4	19.1555
**006880_007**	Strawberries - Class	Serum	10	0.2	0.421637
**006881_007**	Strawberries - Class	Serum	10	0.163	0.123473
**006887_007**	Mango - Class Mango - Concentration	Serum	10	0.122	0.041846
**011238_010**	Vanillylmandelic acid (creatinine correction)	urine	10	3.35	0.966379
**011240_010**	creatinine concentration	urine	10	108.873	83.58942
**011358_100**	pH	Unspecified	10	7.3655	0.051752
**011363_100**	SBE	Unspecified	10	1.15	4.178051
**011364_100**	O2CT	Unspecified	10	10.1	5.351012
**011365_100**	O2SAT	Unspecified	10	69.08	29.73874
**011367_100**	Potassium	Unspecified	10	3.92	0.315524
**011369_100**	Calcium ion	Unspecified	10	2.244	0.090823
**011370_100**	Hemoglobin	Unspecified	10	10.27	1.801882
**001573_040**	Cyclic sideroblast	bone marrow	9	0.111111	0.333333
**002709_055**	eosinophil	peritoneal dialysis fluid	9	0.888889	0.650854

**005462_007**	SPan-1 antigen	Serum	9	21.88889	18.503
**005869_007**	Measles HI	Serum	9	34.66667	42.33202
**006000_041**	Varicella Herpes Zoster IgG EIA	spinal fluid	9	0.327778	0.182331
**006004_041**	Varicella/Herpes Zoster IgM EIA	spinal fluid	9	0.21	0.078262
**006425_007**	Rye concentration	Serum	9	0.105556	0.016667
**006440_007**	Sesame - Concentration	Serum	9	0.298889	0.276561
**011277_020**	Calprotectin in stool	stool	9	210.1111	570.1512
**011359_100**	Carbon dioxide partial pressure	Unspecified	9	44.85556	6.781429
**011368_100**	Chlor	Unspecified	9	109.6667	3.807887
**011371_100**	COHb	Unspecified	9	2.088889	0.838815
**011372_100**	FMetHb	Unspecified	9	0.755556	0.343188
**011373_100**	Blood glucose	Unspecified	9	113.1111	19.56045
**000003_007**	albumin-total globulin ratio	Serum	8	1.18375	0.363473
**001574_040**	Total sideroblasts	bone marrow	8	43.875	5.718079
**002673_045**	creatinine	pleural fluid	8	1.14	0.245706
**002708_047**	lymphocyte	Pericardial fluid	8	12.1875	12.21222
**002711_047**	Other blood cells	Pericardial fluid	8	28.625	37.42111
**005138_008**	Factor V activity	Plasma	8	114.875	37.16541
**005416_007**	Prealbumin	Serum	8	15.8875	2.396687
**005474_007**	Primidone	Serum	8	7.1375	0.489716
**005676_011**	Urinary copper	Fresh Urine	8	15.25	5.444525
**005739_008**	Alpha 2 plasmin inhibitor	Plasma	8	120	2.13809
**005800_046**	Ascites-CA125	ascites	8	1497.875	823.6481
**010128_007**	Teicoplanin	Serum	8	26.475	8.784361
**011123_007**	SOUCARNITINE	Serum	8	49.4125	22.11085
**011124_007**	Eucarnitine	Serum	8	34.875	15.46108
**011125_007**	Acylcarnitine	Serum	8	14.5375	7.790826
**011239_010**	Vanillylmandelic acid concentration	urine	8	2.425	1.022252
**011357_100**	Body temperature	Unspecified	8	36.975	0.070711
**011360_100**	Oxygen partial pressure	Unspecified	8	42.65	26.64893
**011366_100**	Sodium	Unspecified	8	138.375	2.065879
**011374_100**	Lactate	Unspecified	8	1.0125	0.527629
**001001_062**	White Blood Cell Count	Stem cell	7	185.0143	25.76344
**001003_062**	hemoglobin	Stem cell	7	0.257143	0.053452
**001013_062**	promyelocytes	Stem cell	7	4.285714	2.098752
**001014_062**	Bone marrow cells	Stem cell	7	9.5	5.515131
**001021_062**	lymphocyte	Stem cell	7	35.42857	18.44683
**001220_062**	Neutrophil count	Stem cell	7	14.56286	14.50458
**001223_062**	Lymphocytes	Stem cell	7	68.22857	37.61923
**001224_062**	Monocyte Count	Stem cell	7	69.93714	15.14248
**001228_062**	Promyelocyte count	Stem cell	7	7.591429	2.984138
**001229_062**	Myelocyte count	Stem cell	7	17.20286	9.528651

**002710_055**	basophil	peritoneal dialysis fluid	7	0.857143	0.475595
**005295_007**	Lipoprotein fraction alpha	Serum	7	23.85714	7.81939
**005296_007**	lipoprotein fraction PRE beta	Serum	7	32.28571	11.44136
**005297_007**	Lipoprotein fraction beta	Serum	7	38.57143	9.554356
**005300_007**	lipoprotein fraction chylomicron	Serum	7	5.285714	6.701102
**005334_007**	Total bile acids	Serum	7	3.9	1.949359
**005657_010**	Myoglobin quantification	urine	7	78.44286	153.622
**005719_012**	Free cortisol	urine collection	7	41.02857	16.55755
**005789_046**	Adenosine deaminase	ascites	7	13.2	9.821745
**005792_046**	Ascites - CEA	ascites	7	4.814286	7.605794
**005798_046**	Ascites-CA19-9	ascites	7	72.4	164.2681
**005923_007**	Coxsackie A2 NT	Serum	7	594.2857	993.1226
**005927_007**	Coxsackie A6 NT	Serum	7	8	5.656854
**006416_007**	alpha-lactalbumin Concentration	Serum	7	0.1	1.50E-17
**006485_007**	Salmon Salmon concentration	Serum	7	0.1	1.50E-17
**006830_007**	Coconut Concentration	Serum	7	0.1	1.50E-17
**006853_007**	Carrot - Class	Serum	7	1.714286	1.704336
**006957_007**	Ovomucoid	Serum	7	0.857143	1.069045
**006958_007**	Ovomucoid Concentration	Serum	7	0.771429	1.007694
**006973_007**	Selenium	Serum	7	68.85714	5.080307
**007381_007**	Yams Class	Serum	7	0.714286	0.95119
**009073_001**	T cell Absolute number	Blood	7	81.71429	5.18698
**009074_001**	B cell Absolute number	Blood	7	7.571429	2.070197
**011180_007**	Anti SS-A (immunodiffusion assay)	Serum	7	10.71429	11.32423
**011202_007**	Estradiol	Serum	7	290.7143	106.3839
**011203_007**	Progesterone	Serum	7	7.695714	9.598684
**000001_007**	Total protein	Serum	6	7.766667	0.388158
**001002_062**	Red Blood Cell Count	Stem cell	6	0.113333	0.060222
**001004_062**	Hematocrit	Stem cell	6	1.133333	0.287518
**001009_062**	platelet count	Stem cell	6	1021	289.2307
**001015_062**	Back myelocyte	Stem cell	6	3.25	2.734045
**001018_062**	nucleus segmental neutrophil	Stem cell	6	4.583333	3.455672
**001022_062**	monocyte	Stem cell	6	34.5	5.576737
**001226_062**	Nucleus Minorum Neutrophils	Stem cell	6	8.56	6.53021
**001230_062**	Post myelocyte count	Stem cell	6	6.181667	5.544945
**001566_040**	Result 2	bone marrow	6	0.766667	1.120119
**002672_045**	Urea nitrogen	pleural fluid	6	16.13333	4.737369
**002675_085**	Total bilirubin	Other	6	1.716667	2.184872
**002677_047**	Neutral Fat	Pericardial fluid	6	29.66667	7.31209
**002695_045**	Protein fraction	pleural fluid	6	1.48	0.598665
**002696_045**	albumin	pleural fluid	6	57.6	10.59925
**002697_045**	alpha 1 globulin	pleural fluid	6	4.833333	1.838115

**002698_045**	alpha 2 globulin	pleural fluid	6	5.5	0.887694
**002700_045**	gamma globulin	pleural fluid	6	22.75	10.05719
**002714_047**	pH	Pericardial fluid	6	7.1	0.109545
**002851_047**	Nucleated cells	Pericardial fluid	6	1345	1687.51
**002853_085**	monocyte	Other	6	0.833333	0.408248
**005137_008**	Factor II activity	Plasma	6	96.83333	28.35783
**005374_007**	Vitamin E	Serum	6	1.51	0.853628
**005376_007**	Retinol-binding protein	Serum	6	3.8	1.885736
**005638_007**	anti ss-DNAIgG antibody	Serum	6	6.85	2.896032
**005653_010**	C peptide	urine	6	41.8	25.51462
**005662_010**	Transferrin	urine	6	11952.58	11980.11
**005663_010**	Creatinine	urine	6	118.5967	110.1973
**005664_010**	Creatinine Corrected value	urine	6	150.8667	105.302
**005679_010**	Coproporphyrin Determination	urine	6	139.1667	51.78578
**005724_010**	Uroporphyrin	urine	6	3272.667	1190.919
**005917_007**	Influenza A (H3N2)	Serum	6	111.6667	74.94442
**005931_007**	Coxsackie Model A10 NT	Serum	6	46	102.8786
**006854_007**	Carrots - Concentration	Serum	6	2.58	3.31132
**006862_007**	Celery - Class	Serum	6	1.166667	1.834848
**006875_007**	Orange - Class Orange - Concentration	Serum	6	0.1	1.52E-17
**007161_062**	CD34	Stem cell	6	1.032917	0.117709
**007342_007**	Clams Concentration	Serum	6	0.1	1.52E-17
**007382_007**	Yams Concentration	Serum	6	0.341667	0.342895
**007391_007**	Walnuts	Serum	6	0.166667	0.408248
**007392_007**	Walnuts (food) Concentration	Serum	6	0.203333	0.196943
**011175_007**	Anti-p53 antibody	Serum	6	71.30333	106.7525
**011237_012**	Vanillylmandelic acid fixed dose	urine collection	6	1.55	0.61563
**011361_100**	HCO3	Unspecified	6	25.81667	4.346685
**011362_100**	Actual base excess	Unspecified	6	1	4.122621
**001010_062**	Mean platelet volume	Stem cell	5	10.1	1.079352
**001017_062**	rod-shaped neutrophil	Stem cell	5	2.7	1.788854
**001025_062**	Hemogram 1	Stem cell	5	1.2	0.273861
**001225_062**	Rod-shaped nucleus neutrophil count	Stem cell	5	5.01	3.370185
**001232_062**	Hemogram 1#	Stem cell	5	2.194	0.804692
**001233_002**	Hemogram item 2#	venous blood	5	0.41	0.342272
**002669_085**	Chlor	Other	5	105.2	5.80517
**002686_085**	Pancreatic amylase	Other	5	61.2	71.18778
**002699_045**	beta globulin	pleural fluid	5	9.92	0.739594
**002710_085**	basophil	Other	5	0.6	0.223607
**005160_010**	IgG	urine	5	12.92	25.20559
**005191_007**	Insulin 180 min	Serum	5	28.04	23.98235
**005366_007**	Vitamin A	Serum	5	42.9	14.94858

**005453_007**	Total PSA	Serum	5	3.668	2.546973
**005454_007**	Free PSA	Serum	5	0.55	0.193778
**005791_045**	Pleural fluid-CEA	pleural fluid	5	27.32	35.27707
**005916_007**	Influenza A (H1N1)	Serum	5	88	130.0769
**006350_007**	Wheat	Serum	5	0.13	0.067082
**006449_007**	Potatoes - Class Potatoes - Concentration	Serum	5	0.128	0.030332
**006863_007**	Celery - Concentration	Serum	5	1.92	3.975292
**006889_007**	Avocado - Class	Serum	5	0.2	0.447214
**006890_007**	Avocado - Concentration	Serum	5	0.186	0.192302
**007081_007**	Ikura	Serum	5	0.2	0.447214
**007082_007**	Salmon roe Concentration	Serum	5	0.18	0.15248
**010169_007**	Anti-LKM-1 antibody	Serum	5	7.56	3.102096
**011143_060**	Detection type 4	fluid to be wiped out	5	57.8	6.83374
**011236_012**	Vanillylmandelic acid titer daily	urine collection	5	2.76	0.250998
**011376_100**	FiO2	Unspecified	5	21.6	1.341641
**000142_001**	Methotrexate	Blood	4	0.12825	0.106828
**001001_040**	White Blood Cell Count	bone marrow	4	9.925	1.666083
**001002_040**	Red Blood Cell Count	bone marrow	4	3.365	0.339657
**001003_040**	hemoglobin	bone marrow	4	9.975	1.209339
**001072_008**	Prothrombin time	Plasma	4	12.175	2.132878
**001073_008**	Prothrombin time ratio	Plasma	4	1.01	0.174929
**001074_008**	Prothrombin International Normalized Ratio	Plasma	4	1.01	0.174929
**001109_008**	Prothrombin time activity	Plasma	4	102.75	29.90401
**002667_085**	Sodium	Other	4	141.75	2.986079
**002668_085**	Potassium	Other	4	4.1	0.141421
**002673_085**	Creatinine	Other	4	5.3625	9.40579
**002676_047**	Cholesterol	Pericardial fluid	4	81.75	11.14675
**002677_055**	Neutral Fat	peritoneal dialysis fluid	4	5.75	3.5
**002713_085**	Specific gravity	Other	4	1.02075	0.016256
**003006_001**	Blood glucose 180 min value	Blood	4	90.25	25.86342
**005277_007**	1,5-Anhydroglucitol	Serum	4	8.625	4.361479
**005452_007**	PSA-F/T ratio	Serum	4	0.1775	0.027538
**005485_007**	Lidocaine	Serum	4	3.575	3.629853
**005929_007**	Coxsackie Model A9 NT	Serum	4	22	28
**006190_007**	Cortisol 90 min value	Serum	4	12.45	8.118091
**006434_007**	Corn Concentration	Serum	4	0.155	0.064031
**006883_007**	Pears - Class	Serum	4	1.25	1.5
**006902_007**	Cod Cod concentration	Serum	4	0.1575	0.115
**011200_007**	Luteinizing hormone	Serum	4	2.575	1.184272
**011201_007**	Follicle-stimulating hormone	Serum	4	14.45	5.640626
**011375_100**	mOsm	Unspecified	4	284.3	2.054264
**000011_007**	LDL-cholesterol	Serum	3	140	11.53256

**000012_007**	HDL-cholesterol	Serum	3	60	4
**000025_007**	Creatine kinase	Serum	3	80.66667	10.11599
**000035_007**	Sodium	Serum	3	140.3333	0.57735
**000036_007**	Potassium	Serum	3	4.233333	0.11547
**000037_007**	Chlor	Serum	3	105.6667	0.57735
**001001_047**	White Blood Cell Count	Pericardial fluid	3	10.13333	13.82835
**001002_047**	Red Blood Cell Count	Pericardial fluid	3	1.946667	1.747493
**001003_047**	hemoglobin	Pericardial fluid	3	5.6	4.915282
**001004_040**	hematocrit	bone marrow	3	28.26667	1.882374
**001004_047**	hematocrit	Pericardial fluid	3	18.13333	15.7754
**001020_062**	basophil	Stem cell	3	0.833333	0.57735
**001027_002**	Hemogram 2	venous blood	3	10.33333	0.288675
**001075_008**	Activated partial thromboplastin time	Plasma	3	64.6	59.67236
**001078_008**	Fibrinogen	Plasma	3	574.3333	101.3723
**001079_008**	Antithrombin III	Plasma	3	87	27.87472
**001082_008**	D-dimer	Plasma	3	3.133333	1.616581
**001222_062**	Basophil Count	Stem cell	3	1.47	0.995942
**002591_012**	Microalbumin	urine collection	3	71.8	81.19477
**002592_012**	Microalbumin creatinine ratio	urine collection	3	144.4333	191.6347
**002593_012**	Microalbumin 24 hours	urine collection	3	162.7333	218.911
**002667_047**	Sodium	Pericardial fluid	3	126.3333	8.020806
**002668_047**	Potassium	Pericardial fluid	3	4.9	1.126943
**002669_047**	Chlor	Pericardial fluid	3	94.66667	5.859465
**002670_055**	Total Protein	peritoneal dialysis fluid	3	1.1	0.9
**002670_085**	Total protein	Other	3	2.733333	3.326159
**002676_055**	Cholesterol	peritoneal dialysis fluid	3	23	20.07486
**002677_085**	Neutral Fat	Other	3	7	5.196152
**002679_085**	Lactate dehydrogenase	Other	3	576.6667	841.5708
**002692_085**	Glucose	Other	3	25.33333	36.96395
**002695_046**	albumin-total globulin ratio	ascites	3	1.023333	0.238607
**002696_046**	albumin	ascites	3	50.03333	6.21718
**002697_046**	alpha 1 globulin	ascites	3	6.266667	1.950214
**002698_046**	alpha 2 globulin	ascites	3	7.366667	0.85049
**002699_046**	beta globulin	ascites	3	10.8	1.8
**002700_046**	gamma globulin	ascites	3	25.53333	9.757732
**002733_048**	Eosinophils	joint fluid	3	0.833333	0.288675
**005025_007**	Prostate acid phosphatase	Serum	3	1.066667	0.152753
**005139_008**	Factor VII activity	Plasma	3	78.66667	27.59227
**005141_008**	Factor X activity	Plasma	3	79	6.928203
**005258_007**	Trypsin	Serum	3	544	30.11644
**005327_001**	Serotonin	Blood	3	155.0667	55.39146
**005377_001**	Nicotinic acid	Blood	3	4.366667	0.702377

**005417_007**	Alpha 1-microglobulin	Serum	3	44.4	15.41817
**005435_008**	Histamine	Plasma	3	1.163333	0.110151
**005440_007**	KL-6	Serum	3	432.3333	40.51337
**005473_001**	Lithium carbonate	Blood	3	0.933333	0.450925
**005600_007**	Dilution factor	Serum	3	43754.67	75619.04
**005953_007**	Echo Type 9 NT	Serum	3	13.33333	16.16581
**005960_007**	Echo Type 16 NT	Serum	3	5.333333	2.309401
**006162_008**	Adrenocorticotropic Hormone (ACTH) 30 min	Plasma	3	25.86667	22.31285
**006163_008**	Adrenocorticotropic Hormone (ACTH) 60min	Plasma	3	32.9	27.57952
**006164_008**	Adrenocorticotropic Hormone (ACTH) 90min	Plasma	3	24.86667	20.07046
**006242_007**	Methotrexate (96 hr)	Serum	3	0.049667	0.016743
**006371_007**	Guinea pig epithelium	Serum	3	0.1	1.70E-17
**006380_007**	Rabbit epithelium Concentration	Serum	3	0.1	1.70E-17
**006392_007**	Honey bee Concentration	Serum	3	0.1	1.70E-17
**006452_007**	Sweet potatoes Concentration	Serum	3	0.1	1.70E-17
**006455_007**	Bamboo shoots Concentration	Serum	3	0.256667	0.135769
**006458_007**	Grapefruit - Grapefruit - Concentration	Serum	3	0.1	1.70E-17
**006491_007**	Octopus Concentration	Serum	3	0.1	1.70E-17
**006839_007**	Cocoa - concentration	Serum	3	0.1	1.70E-17
**006859_007**	Onion - Class	Serum	3	1	1.732051
**006860_007**	Onion - Concentration	Serum	3	1.233333	1.962991
**006884_007**	Pears - Class - Pears - Concentration	Serum	3	0.343333	0.421466
**006905_007**	Flounder Concentration	Serum	3	0.1	1.70E-17
**007206_008**	Factor VIII inhibitor	Plasma	3	0.533333	0.665833
**007322_007**	Staphylococcus aureus Enterotoxin A Concentration	Serum	3	0.116667	0.028868
**007332_007**	Staphylococcus aureus Enterotoxin B Concentration	Serum	3	0.126667	0.025166
**009017_002**	Erythrocyte surface marker test CD55	venous blood	3	71.7	47.97947
**009052_001**	Lymphocyte Hypervulnerability Assay (PHA)	Blood	3	28466.67	4392.418
**009053_001**	Lymphocyte Hypervulnerability Assay (PHA)	Blood	3	250	120.5778
**011246_012**	5- Hydroxyindole Acetic Acid Daily Dose	urine collection	3	2.533333	0.702377
**011247_012**	5- Hydroxyindole Acetic Acid	urine collection	3	1.433333	0.404145
**000005_007**	creatinine	Serum	2	0.695	0.06364
**000006_007**	Uric acid	Serum	2	5.25	0.353553
**000007_007**	Total bilirubin	Serum	2	0.45	0.070711
**000010_007**	Total cholesterol	Serum	2	212.5	6.363961
**000016_007**	Lactate dehydrogenase	Serum	2	194.5	14.84924
**000018_007**	Alanine aminotransferase	Serum	2	30.5	2.12132
**000019_007**	Aspartate aminotransferase Aspartate aminotransferase alanine aminotransferase	Serum	2	0.95	0.070711

	aspartate aminotransferase alanine aminotransferase Ratio				
**000020_007**	γ-Glutamyltransferase	Serum	2	31	1.414214
**000027_007**	Estimated glomerular filtration rate	Serum	2	63.5	6.22254
**001001_046**	White Blood Cell Count	ascites	2	3.8	1.414214
**001002_046**	Red Blood Cell Count	ascites	2	2.385	0.643467
**001003_046**	hemoglobin	ascites	2	7.3	1.979899
**001004_046**	hematocrit	ascites	2	23.55	7.424621
**001081_008**	Fibrinogen fibrin Degradation products	Plasma	2	3.85	1.484924
**002541_012**	pH	urine collection	2	6.25	1.06066
**002657_041**	Other cell count	spinal fluid	2	18.5	23.33452
**002671_085**	albumin	Other	2	0.35	0.212132
**002675_055**	Total bilirubin	peritoneal dialysis fluid	2	0.15	0.070711
**002679_055**	Lactate dehydrogenase	peritoneal dialysis fluid	2	86	36.76955
**002709_047**	Eosinophil	Pericardial fluid	2	0.75	0.353553
**002713_055**	Specific gravity	peritoneal dialysis fluid	2	1.0145	0.003536
**002714_085**	pH	Other	2	6.9	0.141421
**002853_047**	monocyte	Pericardial fluid	2	1.5	1.414214
**005003_007**	Leucine aminopeptidase	Serum	2	115	41.01219
**005043_007**	Thyroid-stimulating hormone	Serum	2	3.5645	4.349414
**005046_007**	Gentamicin	Serum	2	6.55	8.838835
**005206_007**	Luteinizing Hormone 30 min Value	Serum	2	41.95	35.00179
**005213_007**	Follicle-stimulating hormone 30 min	Serum	2	14.7	1.979899
**005220_007**	Prolactin 30 min	Serum	2	191.095	161.1143
**005221_007**	Prolactin 60 min	Serum	2	110.165	86.18925
**005227_007**	Thyroid-stimulating hormone 30 min value	Serum	2	37.23	12.98248
**005228_007**	Thyroid Stimulating Hormone 60minute value	Serum	2	30.705	11.85818
**005309_007**	Cortisol	Serum	2	12.93	1.951615
**005328_008**	5- Hydroxyindole Acetic acid	Plasma	2	3.65	0.636396
**005332_007**	Phospholipids	Serum	2	263.5	24.74874
**005430_001**	Lead Concentration	Blood	2	1.8	1.131371
**005479_007**	Disopyramide	Serum	2	3.6	1.131371
**005641_007**	anti-ds-DNAIgM antibody	Serum	2	7.5	0.707107
**005660_011**	Lysozyme	Fresh Urine	2	8.35	11.10158
**005751_008**	Factor VIII-like antigen quantitation	Plasma	2	173.5	38.89087
**005924_007**	Coxsackie A3 NT	Serum	2	320	271.529
**005926_007**	Coxsackie A5 NT	Serum	2	80	67.88225
**006244_007**	C-peptide Before loading	Serum	2	1.9	0.565685
**006249_007**	C-peptide 30 min value	Serum	2	3.9	0.282843
**006250_007**	C-peptide at 60 min	Serum	2	5.55	0.919239
**006251_007**	C-peptide 90 min value	Serum	2	7.5	0.707107

**006252_007**	C-peptide at 120 min	Serum	2	8.1	0.989949
**006727_007**	Anisakis mite - class	Serum	2	1	1.414214
**006728_007**	Mites (Acid mites) Concentration	Serum	2	0.92	1.159655
**006731_007**	Pear mite concentration	Serum	2	0.155	0.077782
**006734_007**	Pteromys volvulus concentration	Serum	2	0.2	0.141421
**006812_007**	Mullet Concentration	Serum	2	0.12	0.028284
**006856_007**	Garlic - Class	Serum	2	1	1.414214
**006857_007**	Garlic - Concentration	Serum	2	0.775	0.92631
**006868_007**	Spinach - Class	Serum	2	1	1.414214
**006869_007**	Spinach - Class Spinach - Concentration	Serum	2	1.685	2.241528
**006895_007**	Lobster - Class	Serum	2	1	1.414214
**006896_007**	Lobster - Concentration	Serum	2	0.515	0.586899
**007028_008**	Granulocyte colony-stimulating factor	Plasma	2	69.8	25.73869
**007084_007**	Cod roe	Serum	2	1	1.414214
**007085_007**	Cod Concentration	Serum	2	0.765	0.940452
**007133_001**	NK cell activity	Blood	2	34.5	10.6066
**007291_012**	Free adrenaline	urine collection	2	9.1	8.626703
**007292_012**	Free noratrenaline	urine collection	2	183.3	225.8499
**007293_012**	Free dopamine	urine collection	2	235	63.63961
**009018_002**	Erythrocyte surface marker test CD59	venous blood	2	66.7	47.09331
**009051_001**	Lymphocyte juvenilization test (Con-A)	Blood	2	188.5	79.90307
**010103_007**	NT-proBNP	Serum	2	48.8	3.394113
**010162_007**	Cashew nuts	Serum	2	1	1.414214
**010163_007**	Cashew nuts Concentration	Serum	2	0.495	0.346482
**011144_060**	Detection type 5	fluid to be wiped out	2	62	8.485281
**011177_008**	Vascular endothelial growth factor	Plasma	2	76.95	65.12453
**011388_041**	Albumin	spinal fluid	2	50	38.18377

†: standard deviation
